# Identification and characterization of *Bol.TNL.2,* a key clubroot resistance gene from cabbage, in *Arabidopsis* and *Brassica napus* L.

**DOI:** 10.1093/hr/uhaf208

**Published:** 2025-08-19

**Authors:** Yiji Shi, Ke Xu, Feixue Zhao, Shunjun Bao, Kai Wang, Lin Zheng, Mingjiao Lu, Weinan Sun, Xiaoyan Li, Aixia Xu, Hongyu Sha, Tianye Zhang, Jiapeng Wu, Sheng Liu, Keqi Li, Zhen Huang

**Affiliations:** State Key Laboratory of Crop Stress Biology for Arid Areas, College of Agronomy, Northwest A&F University, Yangling, Shaanxi 712100, China; State Key Laboratory of Crop Stress Biology for Arid Areas, College of Agronomy, Northwest A&F University, Yangling, Shaanxi 712100, China; State Key Laboratory of Crop Stress Biology for Arid Areas, College of Agronomy, Northwest A&F University, Yangling, Shaanxi 712100, China; State Key Laboratory of Crop Stress Biology for Arid Areas, College of Agronomy, Northwest A&F University, Yangling, Shaanxi 712100, China; State Key Laboratory of Crop Stress Biology for Arid Areas, College of Agronomy, Northwest A&F University, Yangling, Shaanxi 712100, China; State Key Laboratory of Crop Stress Biology for Arid Areas, College of Agronomy, Northwest A&F University, Yangling, Shaanxi 712100, China; State Key Laboratory of Crop Stress Biology for Arid Areas, College of Agronomy, Northwest A&F University, Yangling, Shaanxi 712100, China; State Key Laboratory of Crop Stress Biology for Arid Areas, College of Agronomy, Northwest A&F University, Yangling, Shaanxi 712100, China; State Key Laboratory of Crop Stress Biology for Arid Areas, College of Agronomy, Northwest A&F University, Yangling, Shaanxi 712100, China; State Key Laboratory of Crop Stress Biology for Arid Areas, College of Agronomy, Northwest A&F University, Yangling, Shaanxi 712100, China; State Key Laboratory of Crop Stress Biology for Arid Areas, College of Agronomy, Northwest A&F University, Yangling, Shaanxi 712100, China; State Key Laboratory of Crop Stress Biology for Arid Areas, College of Agronomy, Northwest A&F University, Yangling, Shaanxi 712100, China; State Key Laboratory of Crop Stress Biology for Arid Areas, College of Agronomy, Northwest A&F University, Yangling, Shaanxi 712100, China; Key Laboratory of Biology and Genetic Improvement of Oil Crops, Oil Crops Research Institute of the Chinese Academy of Agricultural Sciences, Ministry of Agriculture, Wuhan 430062, China; State Key Laboratory of Crop Stress Biology for Arid Areas, College of Agronomy, Northwest A&F University, Yangling, Shaanxi 712100, China; State Key Laboratory of Crop Stress Biology for Arid Areas, College of Agronomy, Northwest A&F University, Yangling, Shaanxi 712100, China

## Abstract

Clubroot is a devastating soil-borne disease that parasitizes cruciferous crops, posing a severe threat to rapeseed production. To date, no clubroot-resistant (CR) genes have been successfully cloned in cabbage (*Brassica oleracea*). This study aimed to identify CR genes and elucidate the molecular mechanisms underlying clubroot resistance in *B. oleracea*. A BC_1_ mapping population was developed from a cross between CR cabbage W12 and clubroot-susceptible cabbage Z5. A major CR locus, *Bol.CR7.1*, was identified on chromosome C07 by Bulk Segregant Analysis. Subsequently, the *Bol.CR7.1* was fine-mapped to a 170.2-kb interval using linkage analysis. Two candidate genes, *Bol.TNL.2* and *Bol.TNL.3*, exhibiting sequence variations between the parents were induced upon *Plasmodiophora brassicae* infection. Overexpression of *Bol.TNL.2^W^* (CR cabbage W12) in *Arabidopsis* and rapeseed significantly reduced the disease index compared to the wild type (WT) after *P. brassicae* inoculation. In contrast, plants overexpressing *Bol.TNL.2^Z^* (the susceptible cabbage Z5), *Bol.TNL.3^W^*, and *Bol.TNL.3^Z^* exhibited symptoms comparable to those of WT, indicating that *Bol.TNL.2* is a CR gene. RNA-seq analysis revealed that *Bol.TNL.2* may mediate resistance to *P. brassicae* by modulating pathways related to reactive oxygen species, cell wall metabolism and modification, as well as secondary metabolite synthesis. In addition, long noncoding RNAs were found to play a significant role in regulating gene expression associated with *P. brassicae* interaction. This study broadens the repertoire of CR genes, offering a solid foundation for breeding CR cruciferous crops. Additionally, it provides novel insights into resistance mechanisms in response to *P. brassicae* infection in *B. oleracea*.

## Introduction

In recent years, clubroot caused by *Plasmodiophora brassicae* has attracted significant attention due to its devastating impact on cruciferous crops, resulting in yield losses of at least 10%–15% and, in severe cases, complete crop failure [1, 2]. With agricultural practices such as irrigation, the affected area of clubroot has expanded annually, with the pathogen now detected in more than 60 countries and regions [3]. Clubroot symptoms include swollen roots forming galls, which impair the host’s ability to absorb water and nutrients, leading to stunted growth, wilting, and ultimately death. The infection process involves two stages: the first involves primary zoospore infection of root hairs and epidermal cells, while the second involves secondary zoospores released from root hairs, which further infect the cortex [4].

The most cost-effective method for controlling clubroot is the development of resistant varieties. However, the singularity of resistance loci leads to the loss of resistance in resistant lines. For instance, ‘Mendel’, an introduced clubroot-resistant (CR) variety of *Brassica napus*, began to lose its resistance after being cultivated in Canada for several years [3]. Therefore, it is necessary to identify new resistance genes and develop varieties with multiple resistance loci. To date, more than 20 loci associated with clubroot resistance have been identified in *Brassica rapa*. Among these, chromosome A03 harbors the highest number of loci, including *Crr3a*, *Rcr1*, *Rcr2*, *Rcr4*, *Rcr5*, *Rcr8*, *Rcr9*, *PbBa3.1*, *PbBa3.2*, *PbBa3.3*, *CRq*, *CRk*, *CRd*, *CRb*, *CRa*, *CRA3.7*, *BraA3P5X.Cra/bKato1.1*, and *BraA3P5X.Cra/bKato1.2* [5–19]. Chromosome A08 follows with a considerable number of reported loci, such as *Crr1*, *Crr5*, *Rcr3*, *Cr4Ba8.1*, *PbBa8.1*, and *CRA8.1* [10, 15, 16, 20, 21]. Additionally, resistance loci have been mapped to other chromosomes, including A01 (*Crr2*, *Cr4Ba1.1*, *PbBa1.1*), A02 (*CRc*), and A06 (*Crr4*) [9, 10, 15, 16]. Several quantitative trait loci (QTLs) linked to clubroot resistance, including *CRQTL-GN_1*, *Rcr7*, *PCR.II-2*, *PbC9.1*, *qCRc7–1*, *Rcr_C03-1*, and *BolC.Pb9.1*, have been identified in *Brassica oleracea* [22–28]. To date, no CR genes have been cloned in the C subgenome, and their utilization in breeding programs is still restricted. Hitherto, three CR genes, *CRa*, *Crr1a*, and *Rcr1*, encoding TIR-NBS-LRR (TNL) proteins have been isolated in *B. rapa* [6, 11, 29], and the CR gene *BjuA03.BNT1* cloned from *Brassica juncea* has also been shown to encode a TNL protein [30].

Plants possess two primary immune responses mechanisms: the first is activated when surface-localized receptor kinases detect pathogen-associated molecular patterns (PAMPs), initiating PAMP-triggered immunity (PTI). The second mechanism involves intracellular resistance (R) proteins that recognize effectors secreted by pathogens, which suppress PTI, thereby activating effector-triggered immunity (ETI). Typical R proteins are nucleotide-binding, leucine-rich repeat (NLR) receptor proteins [31]. ETI induces a cascade of defense responses, including reactive oxygen species (ROS), transcriptional reprogramming, and cell wall fortification to prevent pathogen invasion. Transcriptomics has been widely utilized to study the interactions between hosts and pathogens. During the early stages of infection, resistant and susceptible *B. rapa* exhibit distinct gene expression patterns. In resistant *B. rapa*, genes related to PTI and ETI are significantly induced, including those involved in calcium ion influx, glucosinolate biosynthesis, cell wall thickening, salicylic acid (SA) homeostasis, chitin metabolism, and pathogenesis-related (PR) pathways [32]. Similarly, transcriptomic analyses of *B. napus* have shown that SA and ethylene signaling play key roles in resisting *P. brassicae* infection [33]. Furthermore, weighted gene co-expression network analysis of two *B. rapa* accessions identified key pathways related to plant–pathogen interactions and hormone signaling, as well as 15 hub genes, including *RIN4* and *IAA16*, which are potentially involved in resistance to *P. brassicae* [34].

As a class of regulatory RNA molecules exceeding 200 nucleotides in length, long noncoding RNAs (lncRNAs) lack protein-coding capacity and modulate gene expression through *cis*- and *trans*-acting pathways, contributing significantly to plant responses to biotic and abiotic stresses [35]. In maize, *cis-NATZmNAC48* negatively regulates *ZmNAC48*, increasing drought sensitivity [36]. In *Arabidopsis thaliana*, *ELENA1* enhances resistance to *Pseudomonas syringae* pv. tomato DC3000 by upregulating *PR1* expression [37]. Summanwar *et al.* [38] identified 530 differentially expressed lncRNAs (DELs) in resistant and susceptible *B. napus*, which target genes involved in phenylpropanoid biosynthetic pathway, plant–pathogen interactions, and carbon and amino acid biosynthesis pathways. In another resistant locus in *B. napus*, 464 DELs were identified, which participate in plant–pathogen interactions and hormone signaling regulation [39]. Similarly, Aceto *et al.* found that lncRNAs target the plant–pathogen interactions pathway in Chinese cabbage [40]. However, the interaction between lncRNAs and *P. brassicae* in *B. oleracea* remains largely unexplored.

In this study, we used Bulk Segregant Analysis (BSA) and fine mapping to locate a major locus, *Bol.CR7.1*, contributing to resistance against *P. brassicae* in *B. oleracea*. Using strand-specific RNA-seq (ssRNA-seq) and qRT-PCR, we identified two CR candidate genes and subsequently validated that *Bol.TNL.2* confers resistance to *P. brassicae* in W12. Additionally, we profiled the mRNA and lncRNA expression spectra of *B. oleracea* at 14 days postinoculation (dpi) and identified potential disease resistance mechanisms. This study provides a theoretical foundation for breeding CR *B. oleracea* and offers valuable insights into the mechanisms underlying clubroot resistance.

## Results

### Phenotypic characterization of parents and BC_1_ population

**Figure 1:**
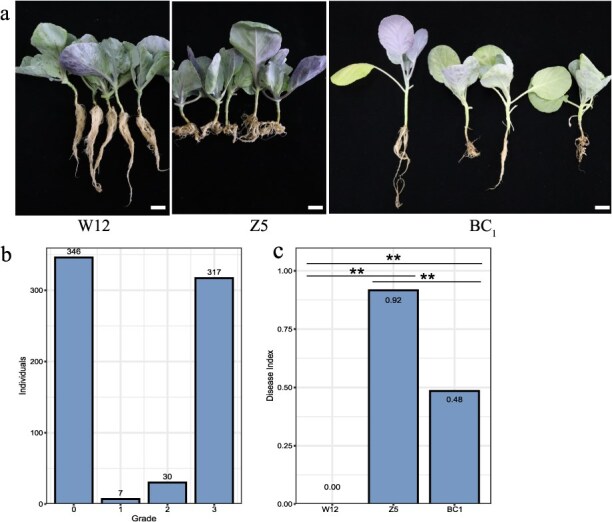
Phenotypic characteristics of plants upon *P. brassicae* inoculation. (a) Phenotypic evaluation of the parental lines and BC₁ progeny 30 days postinoculation (dpi) with *P. brassicae*. Scale bar, 1 cm. (b) Statistical analysis of disease resistance levels in the BC₁ population. (c) Disease index of W12, Z5, and the BC₁ population. Significant differences were determined by *t* test, ^**^P < 0.01.

In order to understand the genetic mechanism underlying resistance to clubroot in cabbage W12, a BC_1_ population was constructed by crossing CR cabbage W12 and the susceptible cabbage Z5 (Fig. 1a). The BC_1_ population was inoculated with *P. brassicae* collected from Mianxian, China. The roots of 700 BC_1_ individuals were examined at 30 days postinoculation (dpi) with *P. brassicae*. The results showed that 346 plants were resistant to clubroot and 354 were susceptible (Fig. 1c), which corresponds to a ratio of approximately 1:1 (*χ^2^* = 0.091, *df* = 1 *P* = .76), suggesting that the resistance to clubroot of W12 is controlled by a major locus.

### Identification of a major CR locus by BSA-seq analysis

To identify the QTL associated with CR in *B. oleracea*, BSA-seq was conducted using a resistant pool, a susceptible pool, and parental lines. A total of 99.79 Gb raw data were filtered to remove low-quality reads, and 92.94 Gb clean data were obtained, achieving a Q30 rate of 92.52%. The clean data of each sample were aligned to the reference genome of *B. oleracea* (HDEM v1.0), and the average mapping rate, sequencing depth, and coverage were 96.27%, 33×, and 94.17%, respectively (Table S1).

**Figure 2:**
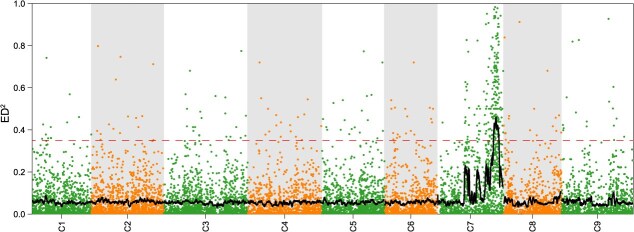
Scatter plot of ED^2^ identifying the candidate region of the *Bol.CR7.1* locus based on BSA-seq data, with the fitted curve generated using a 100-kb sliding window and the dashed line indicating the 99th percentile threshold.

After SNP calling and filtering, a total of 1 752 828 SNPs and 463 226 InDels were identified between the parents, while 572 610 SNPs and 166 955 InDels were found between the resistant and susceptible pools. Among these, 103 602 and 28 229 nonsynonymous mutations resulted in SNPs in the parental lines and the pools, respectively. In order to identify the CR locus, Euclidean distance (ED) was calculated using a 10-kb sliding window analysis. The analysis identified a single region, from 49 550 000 to 54 240 000 bp on chromosome C07. These findings suggest the presence of a major locus offering CR within the interval of 49 550 000 to 54 240 000 bp on chromosome C07, named *Bol.CR7.1* (Fig. 2).

### Fine mapping of *Bol.CR7.1*

**Figure 3:**
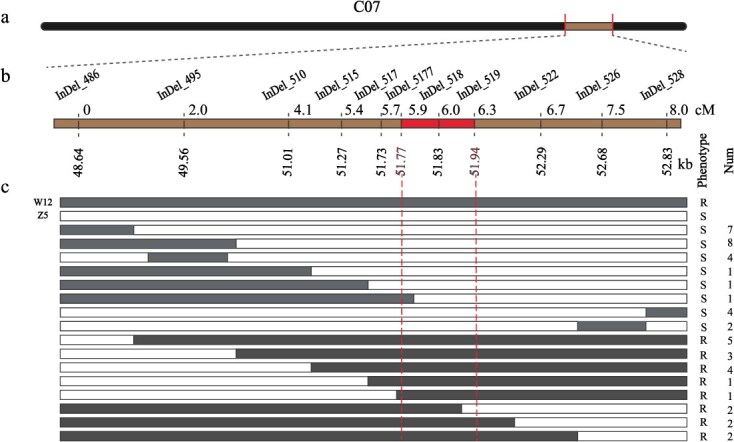
Genetic mapping of the *Bol.CR7.1* locus. (a) C07 chromosome of cabbage. (b) Partial genetic map of *Bol.CR7.1*. The numbers above the bar represent genetic distances (cM), and the highlighted bar represents the region co-segregating with *Bol.CR7.1* in 700 BC_1_ individuals. The numbers under the bar represent physical distances (kb). (c) Haplotypes of 48 recombinant plants screened from 700 BC_1_ individuals delimiting the *Bol.CR7.1* locus to a 170.4-kb region from InDel_5177 to the end of InDel_519. R and S represent resistant and susceptible plants, respectively.

To fine-map the *Bol.CR7.1*, a total of 11 InDels molecular markers were designed based on mutation information between the two parents within the *Bol.CR7.1* region (Table S2). Subsequently, a BC_1_ population consisting of 700 individuals was screened using the InDels markers. A local linkage map was constructed based on the genotypes and phenotypes of individual plants. A total of 48 recombinants were obtained, which were divided into 16 genotypes (Fig. 3c). The results indicated varying degrees of linkage between the markers and the phenotype, which validates the reliability of the BSA results. Ultimately, *Bol.CR7.1* was narrowed down to the interval between markers InDel_5177 (chrC07: 51773364) and InDel_519 (chrC07: 51943569), with a length of 170.2 kb. Among these, InDel_518 co-segregated with the CR gene (Fig. 3b).

### Candidate gene identification

To identify candidate genes for CR, 25 genes within *Bol.CR7.1* locus were annotated using the *Arabidopsis* database. As a result, 19 genes were successfully annotated, among which three genes were identified as disease resistance proteins: *BolC7t45646H* (*Bol.TNL.1*), *BolC7t45647H* (*Bol.TNL.2*), and *BolC7t45649H* (*Bol.TNL.3*; Table S5). Subsequently, Pfamscan was employed to analyze the domain structures of these 25 genes. The results show that Bol.TNL.1 contains both TIR and NB-ARC domains, Bol.TNL.2 contains NB-ARC domains, and Bol.TNL.3 possesses partial TNL structures. None of the remaining genes contain any of the typical domains associated with TNL proteins, including TIR, NB-ARC, or LRR (Table S6). NBS-LRR proteins constitute a large class of R genes in plants that recognize pathogen effectors to activate downstream immune responses [41]. Currently, the cloned CR genes, *CRa* [11], *Crr1a* [6] and *BjuA03.BNT1* [30], all possess TNL domains, suggesting that these three genes may be candidates for CR in *Bol.CR7.1* locus.

To investigate whether *Bol.TNL.1*, *Bol.TNL.2*, and *Bol.TNL.3* respond to infection by *P. brassicae*, we measured the expression levels of these three candidate genes in W12 and Z5 at 7, 14, and 21 dpi. The qRT-PCR results revealed that *Bol.TNL.1* was induced at 7 dpi only in the susceptible cabbage Z5. *Bol.TNL.2* exhibited upregulation at 7 and 21 dpi in both resistant and susceptible lines. In the resistant line W12, *Bol.TNL.2* expression was induced 3.4-fold at 7 dpi and 1.8-fold at 21 dpi. In contrast, in the susceptible line, the gene showed a 2.0-fold increase at 7 dpi and a 1.6-fold increase at 21 dpi. *Bol.TNL.3* was upregulated by 1.8-fold at 21 dpi in the resistant line, and by 2.7- and 3.6-fold at 7 and 21 dpi, respectively, in the susceptible line (Fig. S4). Furthermore, we compared the sequence variations of Bol.TNL.2 and Bol.TNL.3 between the parental lines. The results showed that Bol.TNL.2 exhibited a similarity of only 59.65% (Figs S5 and S6). Notably, *Bol.TNL.2^W^* has a length of 3978 bp and contains 4 exons, whereas *Bol.TNL.2^Z^* is 4101 bp in length and contains 7 exons. Bol.TNL.3 exhibited a similarity of 99.26%, including five nonsynonymous mutations (Fig. S7). Based on these results, *Bol.TNL.2* and *Bol.TNL.3* were identified as CR candidate genes for further investigation.

### Transcriptome analysis reveals the response of cabbage lines with varying resistance to *P. brassicae* inoculation

To investigate the potential pathways response to *P. brassicae* in different CR varieties of *B. oleracea*, ssRNA sequencing was performed on the roots of W12 and Z5 at 14 dpi. A total of 155 Gb raw data were generated and filtered to 148 Gb of high-quality data (Q30 = 93%), with an average mapping rate of 89.63% against the HDEM v1.0 reference genome (Table S4).

**Figure 4:**
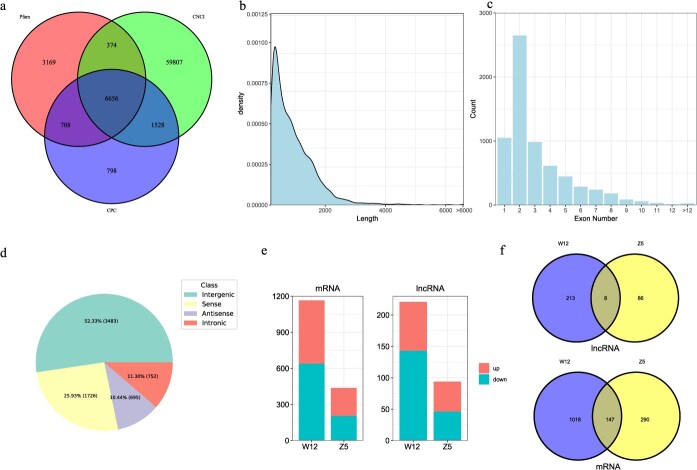
Strand-specific transcriptome analysis. (a) Predicted protein-coding potential of the preliminarily identified lncRNAs using CNCI, Pfam, and CPC. (b) Frequency distribution of the length of lncRNAs. (c) Statistics of exon numbers in lncRNAs. (d) Classification of lncRNAs. (e) Expression changes in mRNAs and lncRNAs at 14 dpi. (f) Differentially expressed genes (DEGs) and differentially expressed lncRNAs (DELs) between resistant and susceptible materials.

Based on the criteria of exon count ≥1, length >200 bp, FPKM ≥0.5, and gffcompare annotation, 26 427 lncRNAs were obtained. Coding potential was assessed using CNCI, CPC, and Pfamscan, by which 9690, 8365, and 10 907 lncRNAs were identified, respectively. The overlap of these predictions identified a total of 6656 lncRNAs (Fig. 4a). The majority of lncRNAs were 237-bp long and had 2 exons (Fig. 4b and c). Based on chromosomal locations, these lncRNAs were classified as intergenic (3483, 52.33%), sense (1726, 25.93%), antisense (695, 10.44%), and intronic (752, 11.30%) (Fig. 4d).

**Figure 5:**
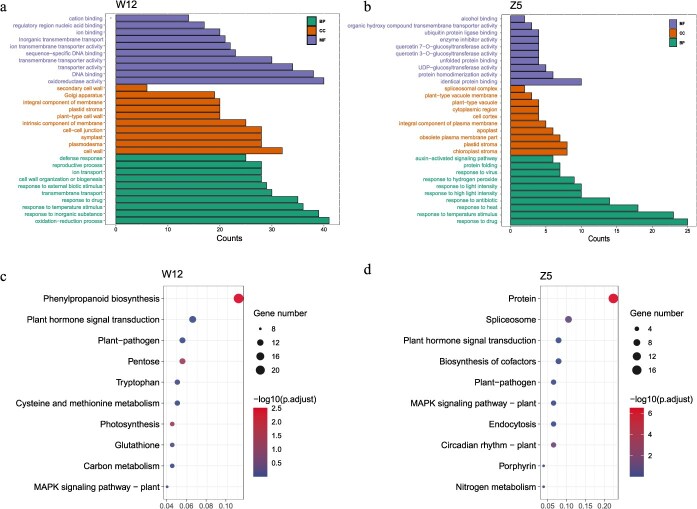
Enrichment analysis of W12 and Z5 at 14 dpi with *P. brassicae*. (a) Top 10 enrichment GO terms of DEGs in W12. (b) Top 10 enrichment GO term of DEGs in Z5. MF, molecular function; CC, cell component; BP, biological process. (c) Top 10 enrichment KEGG pathways of DEGs in W12. (d) Top 10 enrichment KEGG pathways of DEGs in Z5.

Differential expression analysis revealed that, in the comparison between W12-T (inoculation with *P. brassicae*) and W12-CK (control), 526 genes were upregulated and 639 genes were downregulated. In the comparison between Z5-T and Z5-CK, 233 genes were upregulated and 204 were downregulated. In the differential expression lncRNA (DEL) analysis, W12 had 78 upregulated and 143 downregulated DELs (W12-T vs W12-CK), while Z5 had 48 upregulated and 46 downregulated DELs (Z5-T vs Z5-CK). There were 147 commonly DEGs and 8 commonly DELs across these two varieties. The consistency between qRT-PCR and RNA-seq data supports the reliability of the transcriptome analysis (Fig. S1). GO and KEGG enrichment analyses of the DEG were performed for W12 and Z5, respectively. In W12, the enriched terms included oxidoreductase activity, cell wall, and oxidation–reduction process, with KEGG pathways such as phenylpropanoid biosynthesis, plant hormone signal transduction, and plant–pathogen interaction (Fig. 5a and c). By contrast, the DEGs of Z5 were enriched in identical protein binding, chloroplast stroma, and response to drug terms, and enriched in protein processing in the endoplasmic reticulum, spliceosome, and plant hormone signal transduction KEGG pathways (Fig. 5b and d). These results suggest that W12 may activate ROS signaling and cell wall thickening to combat *P. brassicae* infection.

lncRNAs regulate gene expression through both *cis*-acting (upstream/downstream) and *trans*-acting mechanisms. Postinoculation with *P. brassicae*, 51 lncRNAs in W12 regulated 60 *cis*-acting and 101 *trans*-acting target genes, while in Z5, 14 lncRNAs regulated 13 *cis*-acting and 41 *trans*-acting target genes. GO enrichment analysis of these target genes in W12 revealed enrichment in oxidation–reduction processes, immune system processes, postembryonic plant morphogenesis, and innate immune response (Fig. S2). In Z5, no significant enrichment was observed due to the limited number of target genes.

**Figure 6:**
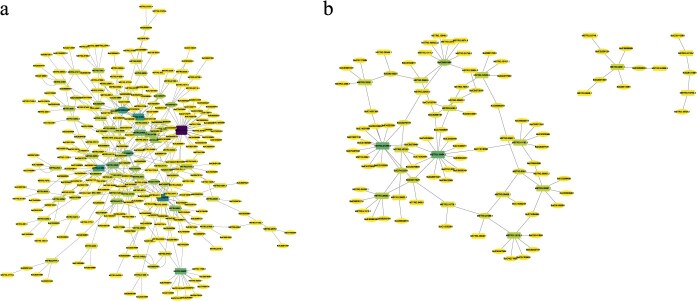
Network regulation diagrams of DELs and target genes in cabbage at 14 dpi with *P. brassicae*. (a) Network diagram of DELs regulating target genes in W12. (b) Network diagram of DELs regulating target genes in Z5. Oval nodes represent target genes, and rectangular nodes represent lncRNAs. Darker colors indicate higher degree values.

Cytoscape was used to construct a network diagram of DELs and target genes in W12 and Z5 at 14 dpi with *P. brassicae*. In W12, *MSTRG.9315.1*, *MSTRG.23714.1*, and *MSTRG.26126.1* had the highest numbers of target genes (39, 23, and 19, respectively, Fig. 6a). In Z5, *MSTRG.21265.3*, *MSTRG.19494.1*, and *MSTRG.12015.1* had the highest numbers of target genes (14, 14, and 7, respectively, Fig. 6b). These genes are potential core lncRNAs involved in interactions with *P. brassicae* and warrant further investigation.

### Functional validation of candidate genes

To further identify the candidate genes, we examined the expression of genes within the *Bol.CR7.1* interval using ssRNA data of W12 and Z5 at 14 dpi with *P. brassicae*. We found that in W12, *Bol.TNL.2* and *BolC7t45653H* (disulfide isomerase-like protein) were induced, whereas in Z5, only *BolC7t45641H* was upregulated (Fig. S3). These findings provide additional evidence that *Bol.TNL.2* is associated with CR in the resistant cabbage W12. Combining the results from qRT-PCR and structural variation analysis, *Bol.TNL.2* and *Bol.TNL.3* were identified as CR candidate genes for further verification.

**Figure 7:**
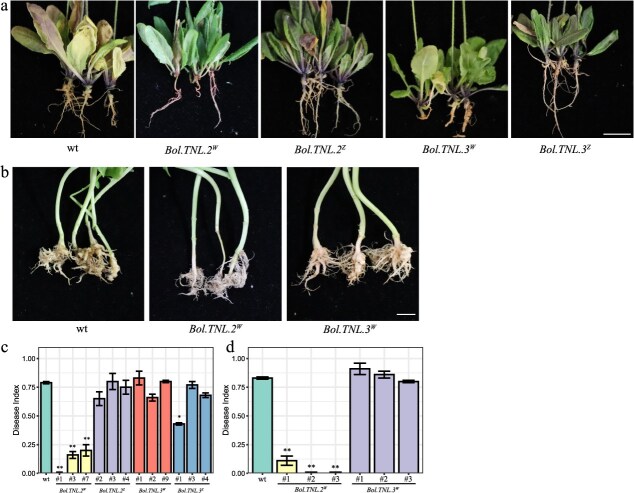
Phenotypic analysis of transgenic plants. (a) Phenotype of transgenic *Arabidopsis thaliana* at 30 dpi with P*. brassicae*. Scale bar, 1 cm. (b) Phenotype of transgenic *B. napus* at 30 dpi with *P. brassicae*. Scale bar, 1 cm. (c) Disease index of transgenic *Arabidopsis thaliana* at 30 dpi with *P. brassicae*. (d) Disease index of transgenic *B. napus* at 30 dpi with *P. brassicae*. The superscript ‘W’ indicates cloning from W12, and the superscript ‘Z’ indicates cloning from Z5.

**Figure 8:**
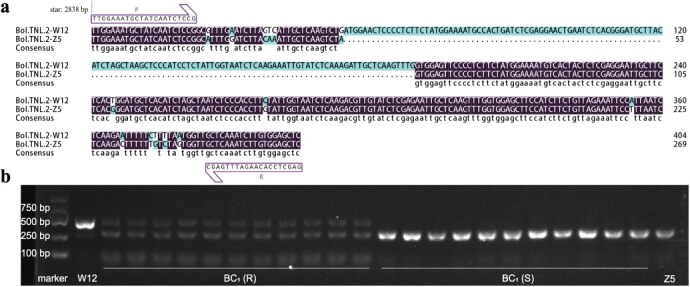
Co-segregation analysis of the clubroot resistance-associated marker Bol.CR7.1_InDel. (a) Schematic diagram of the clubroot resistance-associated InDel marker Bol.CR7.1_InDel. (b) Agarose gel electrophoresis of Bol.CR7.1_InDel in the BC_1_ population, where R indicates resistant individuals and S indicates susceptible individuals.

*Bol.TNL.2^W^* (from W12, resistant), *Bol.TNL.2^Z^* (from Z5, susceptible), *Bol.TNL.3^W^*, and *Bol.TNL.3^Z^* were overexpressed in the roots of *A. thaliana*. The results showed that the average disease index of plants overexpressing *Bol.TNL.2^W^* (from W12, resistant) was 0.05, indicating high resistance to clubroot (Fig. 7a and c). In contrast, the disease index of transgenic plants *Bol.TNL.2^Z^* was 0.89, indicating high susceptibility. The average disease index of the transgenic plants *Bol.TNL.3^W^* and *Bol.TNL.3^Z^* was 0.86 and 0.74, respectively, both resembling the wild type (0.85) (Fig. 7a and c). To further validate these findings, we overexpressed these candidate genes in *B. napus*. The results in *B. napus* are consistent with those observed in *A. thaliana*. Overexpression (OE) of *Bol.TNL.2*^W^ (OE-*Bol.TNL.2*^W^) exhibited no obvious symptoms, with a disease index of 0.04, indicating strong resistance to *P. brassicae* pathotype 4. *Bol.TNL.3^W^* exhibited club-shaped symptoms in the roots, with a disease index of 0.86, similar to the wild type (Fig. 7b and d). These findings align with those observed in *Arabidopsis*. Overall, *Bol.TNL.2^W^* is confirmed as a key gene for resistance to clubroot. Furthermore, based on sequence polymorphisms of *Bol.TNL.2* between the two parental lines, we developed a functional marker (Table S2), which was successfully validated in the BC₁ population (Fig. 8).

### Investigation of *Bol.TNL.2* function through RNA-seq analysis

To further investigate the function of *Bol.TNL.2*, RNA-seq was conducted on *Bol.TNL.2^W^* OE lines and WT plants at 7, 14, and 21 dpi with *P. brassicae* pathotype 4. A total of 28 samples yielded 190.76 Gb of clean data, with an average Q30 score of 96.27%. The alignment rate to the reference genome ranged from 75.48% to 87.80%, with an average of 85.44% (Table S7).

DEGs were identified between the OE lines and WT after *P. brassicae* inoculation at different time points, and a total of 35 341 DEGs were obtained (Fig. S8). At the three inoculation time points, 498, 289, 383, and 692 common DEGs were identified in OE-T (T represents inoculation, Fig. S9a), OE-CK (CK represents mock, Fig. S9b), WT-T (Fig. S9c), and OE-T vs WT-T (Fig. S9d), respectively. These common DEGs may have functional roles at different stages of pathogen infection.

GO enrichment analysis of DEGs between OE-T and WT at these three stages revealed several significant BP, CC, and MF involved in the response to *P. brassicae* infection. At 7 dpi, significant enrichment was observed in processes related to sulfur starvation and aromatic amino acid catabolism, along with enrichment in kinesin complexes and microtubule-associated complexes in OE-T vs WT-T. At 14 dpi, enriched processes included responses to high light intensity and xenobiotic stimuli, with key cellular components such as ER bodies and protein storage vacuoles showing significant enrichment in OE-T vs WT-T. At 21 dpi, enrichment in processes such as monooxygenase activity and xenobiotic transmembrane transporter activity was observed, along with secondary cell wall and casparian strip-related components in OE-T vs WT-T (Fig. S10). KEGG pathway analysis further revealed enrichment in pathways such as plant secondary metabolite biosynthesis and cyanoamino acid metabolism across all time points. (Fig. S11).

Collectively, these results suggest that *Bol.TNL.2* contributes to resistance against *P. brassicae* through modulation of cell wall metabolism, ROS signaling, and the metabolism of bioactive compounds, such as glucosinolates. These findings align with the RNA-seq data from W12 and Z5 at 14 dpi, further confirming the role of *Bol.TNL.2* in the defense response.

## Discussion

Clubroot poses a serious threat to the sustainable production of cruciferous crops such as *B. oleracea*. Developing CR varieties remains the most cost-effective and practical approach to controlling this disease. Over the past two decades, more than 30 CR QTLs have been identified in the A genome. Yang *et al.* [42] integrated 28 published loci into the European fodder turnip ECD04 genome, resulting in 15 integrated QTLs distributed across A03, A08, A06, A01, and A02. In *B. oleracea*, the trait of CR is considered complex and controlled by multiple genes. In the cabbage inbred line GZ84, six QTLs were identified, distributed across chromosomes C04, C07, and C08 [26,43]. In the cabbage cultivar ECD11, two major-effect QTLs and five minor-effect QTLs were identified [27]. Dakouri *et al.* [23] identified a major QTL, *Rcr7*, on C07. Three QTLs were identified on chromosome C09 [25,28], including *BoUGT76C2*, which was validated as the CR gene *BolC.Pb.9.1*. In this study, a major locus, *Bol.CR7.1*, conferring resistance to pathotype 4, was identified through QTL-seq and delimited to a 170.2-kb physical interval from 51 773 364 to 51 943 569 bp on C07 (Fig. 3). Upon alignment to the reference genome (Braol_HDEM_V1.0), *Rcr7* was located in the physical interval from 52 636 076 to 52 922 603 bp on C07. This indicates that *Bol.CR7.1* is different from *Rcr7*.

In the A genome, the CR genes *CRa* [11] and *Crr1a* [6] encode TIR-NBS-LRR (TNL) proteins that are induced upon infection by *P. brassicae*. In plants, NLR proteins function as intracellular immune receptors that recognize pathogen-secreted effectors, triggering an immune response known as ETI [43]. *Bo7g108760* is a TNL gene that serves as a CR candidate gene for *Rcr7* and is induced upon inoculation with the 5X pathotype [23]. In this study, three NLR genes were identified within the *Bol.CR7.1* interval: *Bol.TNL.1*, *Bol.TNL.2*, and *Bol.TNL.3*. Among these, *Bol.TNL.2* displayed upregulated expression at 7 and 21 dpi in both parental lines. *Bol.TNL.3* exhibited upregulated expression at 21 dpi in W12 and at 7 and 21 dpi in Z5. These findings suggest that *Bol.TNL.2* plays a prominent role in conferring clubroot resistance.

Gene structural variations are a key factor contributing to phenotypic differences. For instance, in rice, a variation at amino acid residue 187 in COLD1 leads to differences in cold resistance between indica and japonica cultivars [44]. Similarly, *BoUGT76C2* contains 10 nonsynonymous mutations in CR materials [28]. In this study, we found that Bol.TNL.2 exhibits only 56% similarity between the two parents and contains multiple variations; Bol.TNL.3 has 98% similarity but contains five nonsynonymous mutations, none of which are located in the Tir, NBS, or LRR domains. Upon OE of these genes in *A. thaliana* and *B. napus*, it was found that *Bol.TNL.2^W^* confers resistance to pathotype 4 in both *A. thaliana* and *B. napus*, confirming that *Bol.TNL.2^W^* is a CR gene. These findings provide a valuable resource for breeding CR crops in the Brassicaceae family.

To investigate the mechanisms underlying interactions with *P. brassicae*, strand-specific transcriptome analysis was conducted at 14 dpi to profile lncRNA and mRNA expression in different CR resistant cabbage lines. GO enrichment analysis of DEGs in W12 at 14 dpi revealed that categories such as oxidoreductase activity, cell wall, and oxidation–reduction process were highly enriched. These findings highlight their crucial roles in resistance to clubroot. Previous studies have shown that plant hormones, such as SA, ethylene, and jasmonic acid (JA), along with phenylpropanoid metabolism, are involved in clubroot resistance [32]. Among them, SA plays a crucial role in resistance against *P. brassicae*, whereas the contribution of JA is relatively minor, and its efficacy is insufficient for practical control measures [45]. Additionally, Ferdausi *et al.* [46] found that the synthesis of polyphenols and flavonols is critical for resistance to clubroot. Consistent with these studies, our results also indicate the involvement of phenylpropanoid metabolism and plant hormones in clubroot resistance. ROS are known to play a crucial role in plant responses to biotic stress. For example, in the *Arabidopsis* double mutant *bpl13-2/pire-1*, elevated expression of RBOHD and increased ROS levels led to resistance to bacterial infection [47].

To further elucidate the potential regulatory network of *Bol.TNL.2^W^*, we conducted RNA-seq on *B. napus* overexpressing *Bol.TNL.2^W^* (OE) and WT plants at three critical stages: root hair infection, cortical infection, and protoplast development. GO enrichment analysis of DEGs between OE-T and WT-T at these three stages revealed that the interaction between *P. brassicae* and the host varies significantly at different developmental stages of the pathogen. Common DEGs across all stages were enriched in BP terms related to response to insect, cell wall modification, S-glycoside, and glucosinolate metabolic processes, suggesting that these components play a crucial role in resistance to *P. brassicae*. KEGG enrichment analysis also highlighted the importance of secondary metabolites, such as cyanoamino acids and glucosinolates, in either resisting *P. brassicae* or facilitating its development (Fig. S11). Glucosinolates are considered important secondary metabolites in plant defense against pathogens and pests. In *A. thaliana*, glucosinolates are required for the innate immune response [48]. Studies have shown that the hydrolysis products of glucosinolates exhibit inhibitory effects against pathogens [49]. For instance, indole glucosinolates enhance resistance to *Alternaria brassicicola* in both *Arabidopsis* and *Brassica* crops [50]. However, despite these promising findings, there is currently no direct evidence that glucosinolates, particularly aliphatic or indole derivatives, confer resistance to clubroot. In transcriptome analyses of resistant and susceptible *B. napus*, glucosinolate biosynthesis was found to be involved in regulating clubroot disease [51]. Metabolomic studies of resistant and susceptible Chinese cabbage revealed that resistant varieties accumulated higher levels of glucosinolates [52]. Interestingly, susceptible materials showed elevated levels of indole glucosinolates, which may be linked to the complex relationship between these compounds and IAA synthesis [53]. Aliphatic glucosinolate levels increase in resistant plants after *P. brassicae* inoculation, and exogenous JA treatment promotes their synthesis in *B. napus*, suggesting a potential role in conferring resistance to clubroot disease [54]. Thus, while glucosinolates may play a role in clubroot resistance, further investigation is needed to clarify whether they act directly or indirectly.

The cell wall serves as a natural physical barrier in plants, effectively preventing the invasion and spread of pathogens. Both ssRNA-seq and mRNA-seq results revealed that cell wall metabolism and modification play a significant role against *P. brassicae*. After inoculation, increased lignin accumulation was observed in the CR *B. napus*, while the lignin content decreased in susceptible varieties [55]. This underscores the critical role of the cell wall in defending against *P. brassicae.*

In addition, we identified a total of 6656 lncRNAs in cabbage, among which, 307 were induced by *P. brassicae* infection. Enrichment analysis of their target genes revealed significant enrichment in the oxidation–reduction process, immune system process, and innate immune response. Consistent with previous studies, our results suggest that lncRNAs play a crucial role in regulating key pathways involved in clubroot defense [38,39]. These findings highlight the potential of lncRNAs as pivotal regulators of clubroot resistance.

This study provides valuable insights into the genetic mechanisms underlying clubroot resistance and its implications for Brassicaceae crop protection. The identification of novel loci and *Bol.TNL.2^W^* through combined QTL-seq and RNA-seq approaches offers a robust theoretical foundation for further investigation on the genetic regulation of clubroot resistance, paving the way for improved crop resilience strategies.

## Materials and methods

### Plant materials and culture conditions

Two cabbage varieties, CR W12 and susceptible Z5, were used in this study. A BC_1_ population was developed from a cross between W12 and Z5, using Z5 as the recurrent parent. The planting was initiated by sterilizing the seeds with 75% ethanol for 3 min, followed by three rinses with sterile water. The seeds were then placed on filter paper in petri dishes filled with an adequate amount of sterile water. The petri dishes were incubated at 24°C with 16 hours of light and 20°C with 8 hours of darkness. After germination, the seedlings were transplanted into small pots (6 × 6 × 7 cm) filled with a nutrient soil mixture (substrate/vermiculite/perlite = 3:1:1) and maintained in an incubator for further growth.

### Phenotypic identification

Once the plants reached the three-leaf stage, they were inoculated with *P. brassicae*, which has been identified as pathotype 4 and collected from Mianxian, China. The inoculum was prepared following the method described by Piao *et al.* [56]. The number of resting spores was counted using a hemocytometer, and the final concentration was adjusted to 1 × 10^7^ spores/ml. Each plant was inoculated with 2 ml of the inoculum solution, while the control group was treated with 2 ml of sterile water. At 30 days postinoculation (dpi), plants were uprooted, gently washed to remove soil, and assessed for disease severity using a four-grade scale: Grade 0 (no symptoms), Grade 1 (small galls on lateral roots), Grade 2 (galls on the main root), and Grade 3 (extensive galling on both main and lateral roots). The infection rate and disease index were then calculated following the method of Strelkov *et al.* [57].

### Whole-genome resequencing and Bulk Segregant Analysis

DNA was extracted from leaves using a modified CTAB method [58], and it was used for BSA sequencing after pooling 30 resistant and 30 susceptible plants along with the parents. DNA concentration and integrity were assessed using a NanoDrop One/OneC. Qualified samples were fragmented (~350 bp) by sonication, prepared into sequencing libraries through adapter ligation and PCR amplification, and sequenced on the Illumina HiSeq platform. Adapter sequences, reads with more than 50% bases below Q10 quality, and reads with over 5% N content were removed using Fastp v 0.23.4 [59]. Clean reads were aligned to the reference genome Braol_HDEM_V1.0 (BRAD; http://brassicadb.cn/#/Download/) using BWA-MEM [60]. SAM files were converted to BAM format and sorted using samtools v1.9 [61]. Duplicate reads were removed with Picard (http://broadinstitute.github.io/picard/), and variant calling was conducted using GATK 4.0 [62]. SNPs and InDels were filtered through the GATK VariantFiltration module with the following criteria: cluster window size of 35, cluster threshold of 3, FS ≤60, and QD ≥2.0. To identify regions linked to resistance to CR, the ED algorithm was applied with a sliding window of 1 kb to assess the association between SNPs and traits. The 99th percentile was used as the threshold for QTL detection [63].

### Molecular marker linkage analysis and fine mapping

Within the mapping interval, primers flanking InDels were designed using Primer3 Plus (https://www.primer3plus.com/index.html) based on the resequencing results of the parents. The primer sequences and their positions are listed in Table S2. Polymorphisms were detected using polyacrylamide gel (6%) electrophoresis, and primers showing polymorphisms were used for amplification in the 700 BC_1_ individuals. PCR was performed using Green Taq Mix (Vazyme, Nanjing, China) as per the manufacturer’s instructions. Marker bands consistent with W12 were assigned as A, those matching Z5 as B, heterozygous bands as H, and undefined bands as U. The genetic map was built using JoinMap 4.0 [64] with the Kosambi function to calculate map distances in cM. The annotation information for the 25 candidate genes located within the mapping interval was obtained from the reference genome of *B. oleracea* (Braol_HDEM_V1.0). The functions of these candidate genes were predicted using the Basic Local Alignment Search Tool (BLAST) [65].

### Strand-specific RNA-seq, mRNA-seq, and comparative transcriptome analysis

To investigate the regulatory pathways underlying the response to *P. brassicae*, RNA-seq was performed on the roots of both parental lines at 14 dpi, and on *Bol.TNL.2^W^* OE lines in *B. napus* at 7, 14, and 21 dpi. Three biological replicates were included, with three plants per replicate.

Total RNA was extracted using the RNAprep Pure Plant Kit (TIANGEN, China), and quality was assessed using NanoDrop One/OneC. For ssRNA-seq, rRNA was removed using the Epicentre Ribo-Zero™ Kit, followed by fragmentation to ~150–200 bp. First-strand cDNA was synthesized using random hexamers, followed by second-strand synthesis with DNA Polymerase I and RNase H. After end repair, and adapter ligation, the ssRNA libraries were sequenced on an Illumina HiSeq 4000 platform at Novogene Biotech (Beijing, China). For mRNA-seq, RNA libraries were prepared with the TruSeq RNA Sample Prep Kit (Illumina, USA) and sequenced on the Illumina HiSeq 2500 at BGI.

The low-quality reads were filtered using fastp v0.23.4 [59] with default parameters, and clean data were mapped to reference genome Braol_HDEM_V1.0 or Brana_ZS_HZAU_V1.0 (http://brassicadb.cn/#/Download/). For ssRNA-seq, transcripts were reconstructed using StringTie v2.1.5, with merged transcripts filtered for exon count ≥1, length >200 bp, and FPKM ≥0.5 [66]. Subsequently, the transcripts were selected based on annotation categories (j, I, o, u, or x) using gffcompare v0.12.6 [67]. The selected transcripts were further evaluated for protein-coding potential using CNCI [68], CPC2 [69], and Pfamscan [70] to ultimately determine lncRNAs.

Differential expression analysis of genes (DEGs) and lncRNAs (DELs) was performed using the R package DESeq2 v1.34.0 [71], with |log_2_ Fold Change| > 1 and adjusted *P* value (*P* adjust) <0.05 as thresholds. Gene annotation was conducted using the eggNOG v5.0 database [72]. Gene Ontology (GO) and Kyoto Encyclopedia of Genes and Genomes (KEGG) pathway enrichment analyses were performed using the ClusterProfiler package [73]. To predict the target genes of lncRNAs, we considered protein-coding genes located within 100 kb upstream or downstream of the lncRNAs as *cis*-regulated targets. We used LncTar [74] to predict the *trans*-regulated target genes of lncRNAs. Pearson’s correlation coefficient was used to analyze the correlation between the expression levels of lncRNAs and genes, with a correlation value greater than 0.8 selected for further analysis. The regulation network was built using Cytoscape v3.10.1 [75].

Quantitative real-time PCR (qRT-PCR) was performed using the ChamQ Universal SYBR qPCR Master Mix (Vazyme, Nanjing, China) on a QuantStudio™ 7 Flex Real-Time PCR System (Applied Biosystems, USA). Relative expression levels of the genes were calculated using the 2^−ΔΔCt^ method [76]. Primer sequences are listed in Table S3.

### Plasmid construction and Agrobacterium-mediated transformation

The sequences of *Bol.TNL.2* and *Bol.TNL.3* were predicted using FGENESH (http://www.softberry.com/berry.phtml?topic=fgenesh). Both *Bol.TNL.2* and *Bol.TNL.3* in the parental lines were amplified individually by PCR using Phanta Max Super-Fidelity DNA Polymerase, following the manufacturer’s protocol. Subsequently, the resulting PCR products were ligated with the root-specific promoter pro-*PYK10* into a modified pCAMBIA1300 vector using the ClonExpress MultiS One Step Cloning Kit to construct *PYK10*:*Bol.TNL.2* and *PYK10*:*Bol.TNL.3* [77]. The successfully ligated vector was verified by PCR and then sent to Tsingke (Beijing, China) for Sanger sequencing. Sequence alignment between the two parental alleles was carried out using MAFFT [78]. The *PYK10*:*Bol.TNL.2* and *PYK10*:*Bol.TNL.3* constructs from both parents were introduced into *Agrobacterium tumefaciens* strain GV3101. Wild-type *A. thaliana* ecotype Col-0 was infected using the floral dip method. Transformation of *B. napus* line 1423 was carried out using hypocotyls as explants, as described by Dai *et al.* [79]. Positive transgenic plants were selected with 25 mg/l hygromycin and further verified by PCR using a hyg-specific marker (Table S3).

## Supplementary Material

Web_Material_uhaf208

## Data Availability

All sequencing data have been deposited in the NCBI Sequence Read Archive (SRA). The BSA resequencing data are available under accession number PRJNA1172340, the ssRNA-seq data under PRJNA1172278, and the RNA-seq of OE *B. napus Bol.TNL.2^W^*-OE data under PRJNA1191364.

## References

[1] Chai AL, Xie XW, Shi YX. et al. Research status of clubroot (*Plasmodiophora brassicae*) on cruciferous crops in China. Can J Plant Pathol. 2014;36:142–53

[2] Limei Y, Zhiyuan F, Yangyong Z. et al. Recent advances of disease and stress resistance breeding of cabbage in China. Acta Hortic Sin. 2020;47:1678–88

[3] Strelkov SE, Hwang S-F, Manolii VP. et al. Emergence of new virulence phenotypes of *Plasmodiophora brassicae* on canola (*Brassica napus*) in Alberta, Canada. Eur J Plant Pathol. 2016;145:517–29

[4] Liu L, Qin L, Zhou Z. et al. Refining the life cycle of *Plasmodiophora brassicae*. Phytopathology. 2020;110:1704–12 32407251 10.1094/PHYTO-02-20-0029-R

[5] Chen J, Jing J, Zhan Z. et al. Identification of novel QTLs for isolate-specific partial resistance to *Plasmodiophora brassicae* in *Brassica rapa*. PLoS One. 2013;8:e85307 24376876 10.1371/journal.pone.0085307 PMC3869933

[6] Hatakeyama K, Suwabe K, Tomita RN. et al. Identification and characterization of *Crr1a*, a gene for resistance to clubroot disease (*Plasmodiophora brassicae* Woronin) in *Brassica rapa* L. PLoS One. 2013;8:e54745 23382954 10.1371/journal.pone.0054745 PMC3559844

[7] Huang Z, Peng G, Liu X. et al. Fine mapping of a clubroot resistance gene in Chinese cabbage using SNP markers identified from bulked segregant RNA sequencing. Front Plant Sci. 2017;8:1448 28894454 10.3389/fpls.2017.01448 PMC5581393

[8] Pang W, Fu P, Li X. et al. Identification and mapping of the clubroot resistance gene CRd in Chinese cabbage (*Brassica rapa* ssp. *pekinensis*). Front Plant Sci. 2018;9:653 29868100 10.3389/fpls.2018.00653 PMC5968122

[9] Sakamoto K, Saito A, Hayashida N. et al. Mapping of isolate-specific QTLs for clubroot resistance in Chinese cabbage (*Brassica rapa* L. ssp. *pekinensis*). Theor Appl Genet. 2008;117:759–67 18612625 10.1007/s00122-008-0817-0

[10] Suwabe K, Tsukazaki H, Iketani H. et al. Identification of two loci for resistance to clubroot (*Plasmodiophora brassicae* Woronin) in *Brassica rapa* L. Theor Appl Genet. 2003;107:997–1002 12955203 10.1007/s00122-003-1309-x

[11] Ueno H, Matsumoto E, Aruga D. et al. Molecular characterization of the *Cra* gene conferring clubroot resistance in *Brassica rapa*. Plant Mol Biol. 2012;80:621–9 23054353 10.1007/s11103-012-9971-5

[12] Yu FQ, Zhang XG, Peng G. et al. Genotyping-by-sequencing reveals three qtl for clubroot resistance to six pathotypes of *Plasmodiophora brassicae* in *Brassica rapa*. Sci Rep. 2017;7:4516 28674416 10.1038/s41598-017-04903-2 PMC5495781

[13] Matsumoto E, Yasui C, Ohi M. et al. Linkage analysis of RFLP markers for clubroot resistance and pigmentation in Chinese cabbage (*Brassica rapa* ssp. *pekinensis*). Euphytica. 1998;104:79–86

[14] Chu M, Song T, Falk KC. et al. Fine mapping of *Rcr1* and analyses of its effect on transcriptome patterns during infection by *Plasmodiophora brassicae*. BMC Genomics. 2014;15:1166 25532522 10.1186/1471-2164-15-1166 PMC4326500

[15] Zhan ZX, Jiang YF, Shah ND. et al. Association of clubroot resistance locus *PbBa8.1* with a linkage drag of high erucic acid content in the seed of the European turnip. Front Plant Sci. 2020;11:810 32595684 10.3389/fpls.2020.00810 PMC7301908

[16] Zhang H, Liu X, Zhou J. et al. Identification of clubroot (*Plasmodiophora brassicae*) resistance loci in Chinese cabbage (*Brassica rapa* ssp. *pekinensis*) with recessive character. Genes. 2024;15:274 38540333 10.3390/genes15030274 PMC10970103

[17] Fredua-Agyeman R, Jiang J, Hwang SF. et al. QTL mapping and inheritance of clubroot resistance genes derived from *Brassica rapa* subsp. *rapifera* (ECD 02) reveals resistance loci and distorted segregation ratios in two F_2_ populations of different crosses. Front Plant Sci. 2020;11:899 32719696 10.3389/fpls.2020.00899 PMC7348664

[18] Pang W, Zhang X, Ma Y. et al. Fine mapping and candidate gene analysis of *Cra3.7* conferring clubroot resistance in *Brassica rapa*. Theor Appl Genet. 2022;135:4541–8 36243892 10.1007/s00122-022-04237-2

[19] Wei X, Li J, Zhang X. et al. Fine mapping and functional analysis of major QTL, *Crq* for clubroot resistance in Chinese cabbage (*Brassica rapa* ssp. *pekinensis*). Agronomy. 2022;12:1172

[20] Wang Y, Xiang X, Huang F. et al. Fine mapping of clubroot resistance loci *CRA8.1* and candidate gene analysis in Chinese cabbage (*Brassica rapa* L.). Front Plant Sci. 2022;13:898108 35599882 10.3389/fpls.2022.898108 PMC9121064

[21] Yang S, Wang X, Wang Z. et al. A chromosome-level reference genome facilitates the discovery of clubroot-resistant gene *Crr5* in Chinese cabbage. Hortic Res. 2025;12:uhae338 40046320 10.1093/hr/uhae338 PMC11879649

[22] Lee J, Izzah NK, Choi BS. et al. Genotyping-by-sequencing map permits identification of clubroot resistance QTLs and revision of the reference genome assembly in cabbage (*Brassica oleracea* L.). DNA Res. 2016;23:29–41 26622061 10.1093/dnares/dsv034 PMC4755525

[23] Dakouri A, Zhang XG, Peng G. et al. Analysis of genome-wide variants through bulked segregant RNA sequencing reveals a major gene for resistance to *Plasmodiophora brassicae* in *Brassica oleracea*. Sci Rep. 2018;8:17657 30518770 10.1038/s41598-018-36187-5 PMC6281628

[24] Peng LS, Zhou LL, Li QF. et al. Identification of quantitative trait loci for clubroot resistance in *Brassica oleracea* with the use of *Brassica* SNP microarray. Front Plant Sci. 2018;9:822 29967632 10.3389/fpls.2018.00822 PMC6015909

[25] Farid M, Yang RC, Kebede B. et al. Evaluation of *Brassica oleracea* accessions for resistance to *Plasmodiophora brassicae* and identification of genomic regions associated with resistance. Genome. 2020;63:91–101 31600449 10.1139/gen-2019-0098

[26] Ce F, Mei J, He H. et al. Identification of candidate genes for clubroot-resistance in *Brassica oleracea* using quantitative trait loci-sequencing. Front Plant Sci. 2021;12:703520 34868102 10.3389/fpls.2021.703520 PMC8635040

[27] Karim MM, Yu F. Identification of QTLs for resistance to 10 pathotypes of *Plasmodiophora brassicae* in *Brassica oleracea* cultivar ECD11 through genotyping-by-sequencing. Theor Appl Genet. 2023;136:249 37982891 10.1007/s00122-023-04483-y PMC10661809

[28] Zhang X, Han F, Li Z. et al. Map-based cloning and functional analysis of a major quantitative trait locus, *BolC.Pb9.1*, controlling clubroot resistance in a wild *brassica* relative (*Brassica macrocarpa*). Theor Appl Genet. 2024;137:41 38305900 10.1007/s00122-024-04543-x

[29] Hu H, Zhang Y, Yu F. A CRISPR/Cas9-based vector system enables the fast breeding of selection-marker-free canola with *Rcr1*-rendered clubroot resistance. J Exp Bot. 2024;75:1347–63 37991105 10.1093/jxb/erad471 PMC10901203

[30] Li K, Wang K, Shi Y. et al. *Bjua03.BNT1* plays a positive role in resistance to clubroot disease in resynthesized *Brassica juncea* L. Plant Sci. 2024;349:112268 39313004 10.1016/j.plantsci.2024.112268

[31] Wu Y, Zhou JM. Receptor-like kinases in plant innate immunity. J Integr Plant Biol. 2013;55:1271–86 24308571 10.1111/jipb.12123

[32] Chen J, Pang W, Chen B. et al. Transcriptome analysis of *Brassica rapa* near-isogenic lines carrying clubroot-resistant and -susceptible alleles in response to *Plasmodiophora brassicae* during early infection. Front Plant Sci. 2015;6:1183 26779217 10.3389/fpls.2015.01183 PMC4700149

[33] Zhou Q, Galindo-Gonzalez L, Manolii V. et al. Comparative transcriptome analysis of rutabaga (*Brassica napus*) cultivars indicates activation of salicylic acid and ethylene-mediated defenses in response to *Plasmodiophora brassicae*. Int J Mol Sci. 2020;21:8381 33171675 10.3390/ijms21218381 PMC7664628

[34] Wei X, Zhang Y, Zhao Y. et al. Root transcriptome and metabolome profiling reveal key phytohormone-related genes and pathways involved clubroot resistance in *Brassica rapa* L. Front Plant Sci. 2021;12:759623 34975941 10.3389/fpls.2021.759623 PMC8715091

[35] Ponting CP, Oliver PL, Reik W. Evolution and functions of long noncoding RNAs. Cell. 2009;136:629–41 19239885 10.1016/j.cell.2009.02.006

[36] Mao Y, Xu J, Wang Q. et al. A natural antisense transcript acts as a negative regulator for the maize drought stress response gene ZmNAC48. J Exp Bot. 2021;72:2790–806 33481006 10.1093/jxb/erab023

[37] Seo JS, Sun HX, Park BS. et al. Elf18-induced long-noncoding RNA associates with mediator to enhance expression of innate immune response genes in *Arabidopsis*. Plant Cell. 2017;29:1024–38 28400491 10.1105/tpc.16.00886 PMC5466027

[38] Summanwar A, Basu U, Rahman H. et al. Identification of lncRNAs responsive to infection by *Plasmodiophora brassicae* in clubroot-susceptible and -resistant *Brassica napus* lines carrying resistance introgressed from rutabaga. Mol Plant Microbe In. 2019;32:1360–77 10.1094/MPMI-12-18-0341-R 31090490

[39] Summanwar A, Basu U, Kav NNV. et al. Identification of lncRNAs in response to infection by *Plasmodiophora brassicae* in *Brassica napus* and development of lncRNA-based ssr markers. Genome. 2021;64:547–66 33170735 10.1139/gen-2020-0062

[40] Aceto S, Zhu H, Li X. et al. Integrating long noncoding RNAs and mRNAs expression profiles of response to *Plasmodiophora brassicae* infection in pakchoi (*Brassica campestris* ssp. *Chinensis makino*). PLoS One. 2019;14:e0224927 31805057 10.1371/journal.pone.0224927 PMC6894877

[41] Duxbury Z, Wu CH, Ding P. A comparative overview of the intracellular guardians of plants and animals: NLRs in innate immunity and beyond. Annu Rev Plant Biol. 2021;72:155–84 33689400 10.1146/annurev-arplant-080620-104948

[42] Yang Z, Jiang Y, Gong J. et al. R gene triplication confers European fodder turnip with improved clubroot resistance. Plant Biotechnol J. 2022;20:1502–17 35445530 10.1111/pbi.13827 PMC9342621

[43] Sun Y, Zhu YX, Balint-Kurti PJ. et al. Fine-tuning immunity: players and regulators for plant NLRs. Trends Plant Sci. 2020;25:695–713 32526174 10.1016/j.tplants.2020.02.008

[44] Ma Y, Dai X, Xu Y. et al. Cold1 confers chilling tolerance in rice. Cell. 2015;160:1209–21 25728666 10.1016/j.cell.2015.01.046

[45] Lemarié S, Robert-Seilaniantz A, Lariagon C. et al. Both the jasmonic acid and the salicylic acid pathways contribute to resistance to the biotrophic clubroot agent *Plasmodiophora brassicae* in *Arabidopsis*. Plant Cell Physiol. 2015;56:2158–68 26363358 10.1093/pcp/pcv127

[46] Ferdausi A, Megha S, Liu Y. et al. Metabolomic and transcript level changes reveal the role of polyphenols and flavonols in response to *Plasmodiophora brassicae* infection in *Brassica napus*. J Plant Pathol. 2023;105:1449–64

[47] Lee D, Lal NK, Lin ZD. et al. Regulation of reactive oxygen species during plant immunity through phosphorylation and ubiquitination of RBOHD. Nat Commun. 2020;11:1838 32296066 10.1038/s41467-020-15601-5 PMC7160206

[48] Clay NK, Adio AM, Denoux C. et al. Glucosinolate metabolites required for an *Arabidopsis* innate immune response. Science. 2009;323:95–101 19095898 10.1126/science.1164627 PMC2630859

[49] Bednarek P, Pislewska-Bednarek M, Svatos A. et al. A glucosinolate metabolism pathway in living plant cells mediates broad-spectrum antifungal defense. Science. 2009;323:101–6 19095900 10.1126/science.1163732

[50] Tao H, Miao H, Chen L. et al. *Wrky33*-mediated indolic glucosinolate metabolic pathway confers resistance against *Alternaria brassicicola* in *Arabidopsis* and *brassica* crops. J Integr Plant Biol. 2022;64:1007–19 35257500 10.1111/jipb.13245

[51] Li L, Long Y, Li H. et al. Comparative transcriptome analysis reveals key pathways and hub genes in rapeseed during the early stage of *Plasmodiophora brassicae* infection. Front Genet. 2020;10:1275 32010176 10.3389/fgene.2019.01275 PMC6978740

[52] Lan M, Hu J, Yang H. et al. Phytohormonal and metabolism analysis of *Brassica rapa* L. ssp. *pekinensis* with different resistance during Plasmodiophora brassicae infection. Biocell. 2020;44:751–67

[53] Ludwig-Müller J . Glucosinolates and the clubroot disease: defense compounds or auxin precursors? Phytochem Rev. 2008;8:135–48

[54] Xu L, Yang H, Ren L. et al. Jasmonic acid-mediated aliphatic glucosinolate metabolism is involved in clubroot disease development in *Brassica napus* L. Front Plant Sci. 2018;9:750 29922320 10.3389/fpls.2018.00750 PMC5996939

[55] Tu J, Qin L, Karunakaran C. et al. Lignin accumulation in cell wall plays a role in clubroot resistance. Front Plant Sci. 2024;15:1401265 39109069 10.3389/fpls.2024.1401265 PMC11300216

[56] Piao ZY, Deng YQ, Choi SR. et al. SCAR and CAPS mapping of CRb, a gene conferring resistance to *Plasmodiophora brassicae* in Chinese cabbage (*Brassica rapa* ssp. *pekinensis*). Theor Appl Genet. 2004;108:1458–65 14997298 10.1007/s00122-003-1577-5

[57] Strelkov SE, Tewari JP, Smith-Degenhardt E. Characterization of *Plasmodiophora brassicae* populations from Alberta, Canada. Can J Plant Pathol. 2006;28:467–74

[58] Allen GC, Flores-Vergara MA, Krasynanski S. et al. A modified protocol for rapid DNA isolation from plant tissues using cetyltrimethylammonium bromide. Nat Protoc. 2006;1:2320–5 17406474 10.1038/nprot.2006.384

[59] Chen S, Zhou Y, Chen Y. et al. Fastp: an ultra-fast all-in-one FASTQ preprocessor. Bioinformatics. 2018;34:i884–90 30423086 10.1093/bioinformatics/bty560 PMC6129281

[60] Li H, Durbin R. Fast and accurate short read alignment with Burrows-Wheeler transform. Bioinformatics. 2009;25:1754–60 19451168 10.1093/bioinformatics/btp324 PMC2705234

[61] Li H, Handsaker B, Wysoker A. et al. The sequence alignment/map format and SAMtools. Bioinformatics. 2009;25:2078–9 19505943 10.1093/bioinformatics/btp352 PMC2723002

[62] McKenna A, Hanna M, Banks E. et al. The Genome Analysis Toolkit: a MapReduce framework for analyzing next-generation DNA sequencing data. Genome Res. 2010;20:1297–303 20644199 10.1101/gr.107524.110 PMC2928508

[63] Hill JT, Demarest BL, Bisgrove BW. et al. MMAPPR: mutation mapping analysis pipeline for pooled RNA-seq. Genome Res. 2013;23:687–97 23299975 10.1101/gr.146936.112 PMC3613585

[64] Kosambi DD . The estimation of map distances from recombination values. Ann Eugen. 1943;12:172–5

[65] Camacho C, Coulouris G, Avagyan V. et al. BLAST+: architecture and applications. BMC Bioinformatics. 2009;10:421 20003500 10.1186/1471-2105-10-421 PMC2803857

[66] Pertea M, Pertea GM, Antonescu CM. et al. Stringtie enables improved reconstruction of a transcriptome from RNA-seq reads. Nat Biotechnol. 2015;33:290–5 25690850 10.1038/nbt.3122 PMC4643835

[67] Pertea G, Pertea M. GFF utilities: GFFread and GFFcompare. F1000Research. 2020;9:304 10.12688/f1000research.23297.1 PMC7222033 32489650

[68] Sun L, Luo H, Bu D. et al. Utilizing sequence intrinsic composition to classify protein-coding and long non-coding transcripts. Nucleic Acids Res. 2013;41:e166–6 23892401 10.1093/nar/gkt646 PMC3783192

[69] Kang YJ, Yang DC, Kong L. et al. CPC2: a fast and accurate coding potential calculator based on sequence intrinsic features. Nucleic Acids Res. 2017;45:W12–6 28521017 10.1093/nar/gkx428 PMC5793834

[70] Mistry J, Chuguransky S, Williams L. et al. Pfam: the protein families database in 2021. Nucleic Acids Res. 2020;49:D412–9 10.1093/nar/gkaa913 PMC7779014 33125078

[71] Love MI, Huber W, Anders S. Moderated estimation of fold change and dispersion for RNA-seq data with DESeq2. Genome Biol. 2014;15:550 25516281 10.1186/s13059-014-0550-8 PMC4302049

[72] Cantalapiedra CP, Hernández-Plaza A, Letunic I. et al. EggNOG-mapper v2: functional annotation, orthology assignments, and domain prediction at the metagenomic scale. Mol Biol Evol. 2021;38:5825–9 34597405 10.1093/molbev/msab293 PMC8662613

[73] Yu G, Wang LG, Han Y. et al. ClusterProfiler: an R package for comparing biological themes among gene clusters. Omics: J Integr Biol. 2012;16:284–7 10.1089/omi.2011.0118 PMC3339379 22455463

[74] Li J, Ma W, Zeng P. et al. LncTar: a tool for predicting the RNA targets of long noncoding RNAs. Brief Bioinform. 2015;16:806–12 25524864 10.1093/bib/bbu048

[75] Shannon P, Markiel A, Ozier O. et al. Cytoscape: a software environment for integrated models of biomolecular interaction networks. Genome Res. 2003;13:2498–504 14597658 10.1101/gr.1239303 PMC403769

[76] Livak KJ, Schmittgen TD. Analysis of relative gene expression data using real-time quantitative PCR and the 2^−ΔΔCT^ method. Methods. 2001;25:402–8 11846609 10.1006/meth.2001.1262

[77] Nitz I, Berkefeld H, Puzio PS. et al. *Pyk10*, a seedling and root specific gene and promoter from *Arabidopsis thaliana*. Plant Sci. 2001;161:337–46 11448764 10.1016/s0168-9452(01)00412-5

[78] Katoh K, Standley DM. MAFFT multiple sequence alignment software version 7: improvements in performance and usability. Mol Biol Evol. 2013;30:772–80 23329690 10.1093/molbev/mst010 PMC3603318

[79] Dai C, Li Y, Li L. et al. An efficient *Agrobacterium*-mediated transformation method using hypocotyl as explants for *Brassica napus*. Mol Breed. 2020;40:96

