# Supplementary figures and images for: Identification and characterization of Bol.TNL.2, a key clubroot resistance gene from cabbage, in Arabidopsis and Brassica napus L
Source: Hortic Res. 2025 Aug 19;12(11):uhaf208. doi: 10.1093/hr/uhaf208 (PMC12577853; doi:10.1093/hr/uhaf208)

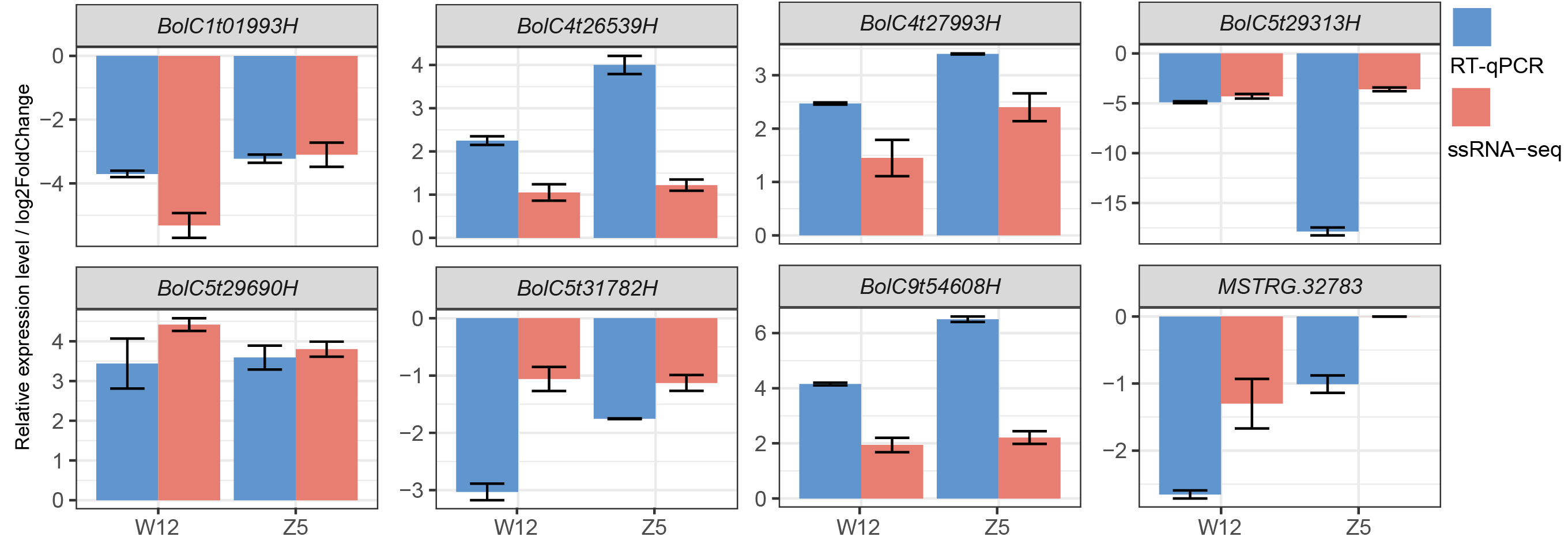

Supplement: Web_Material_uhaf208 [file web_material_uhaf208.zip › FigS1.tif]

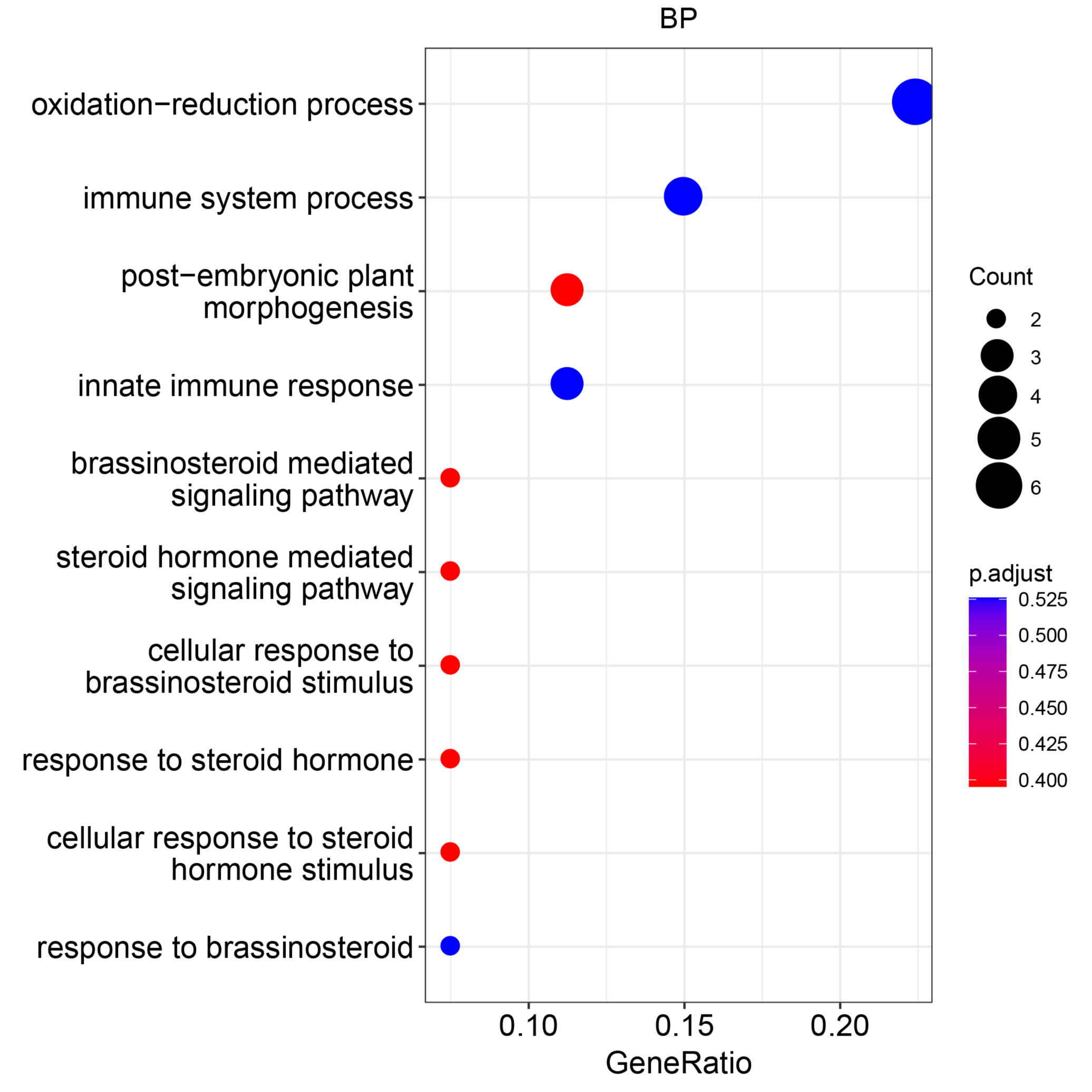

Supplement: Web_Material_uhaf208 [file web_material_uhaf208.zip › FigS2.tif]

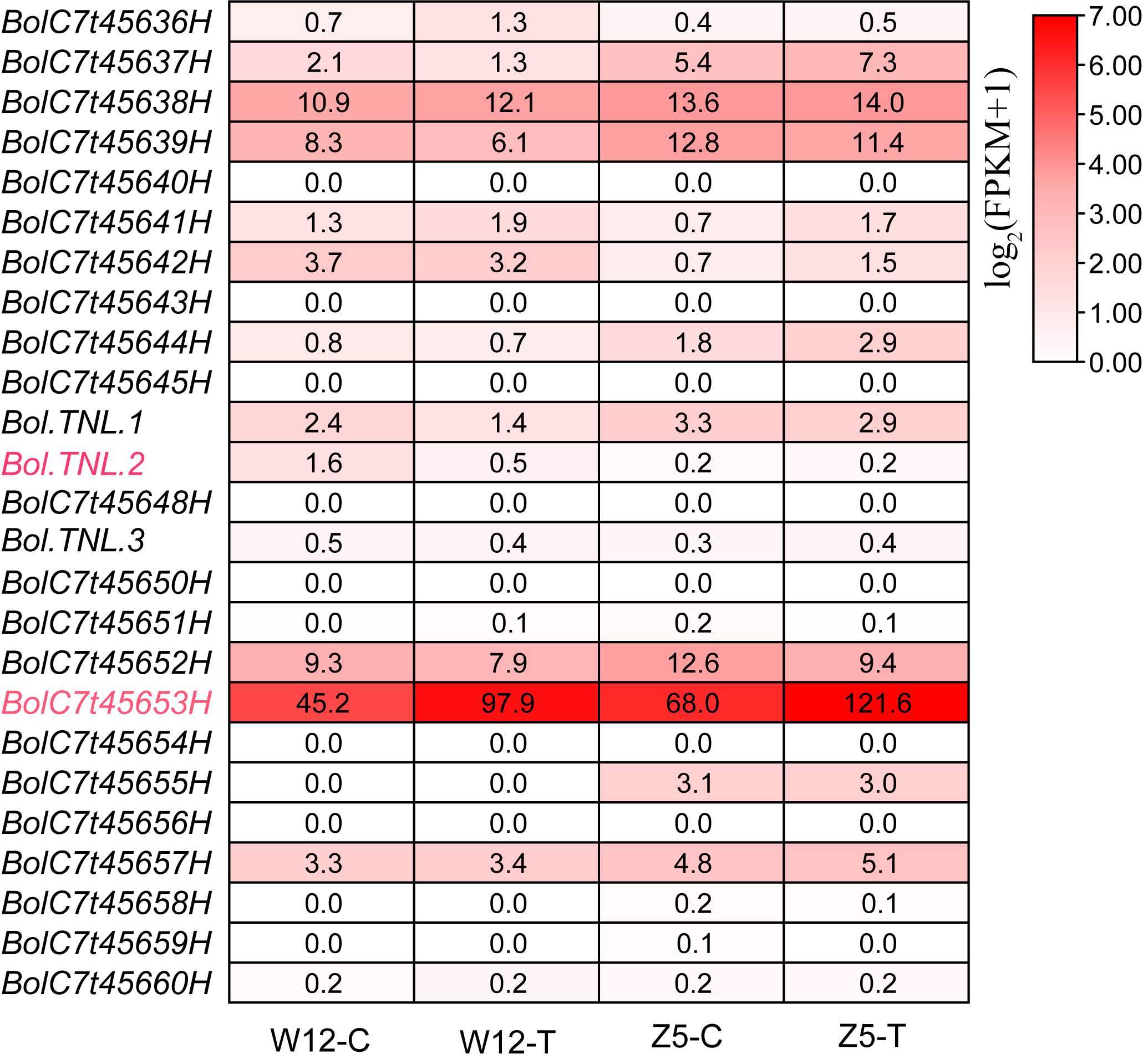

Supplement: Web_Material_uhaf208 [file web_material_uhaf208.zip › FigS3.tif]

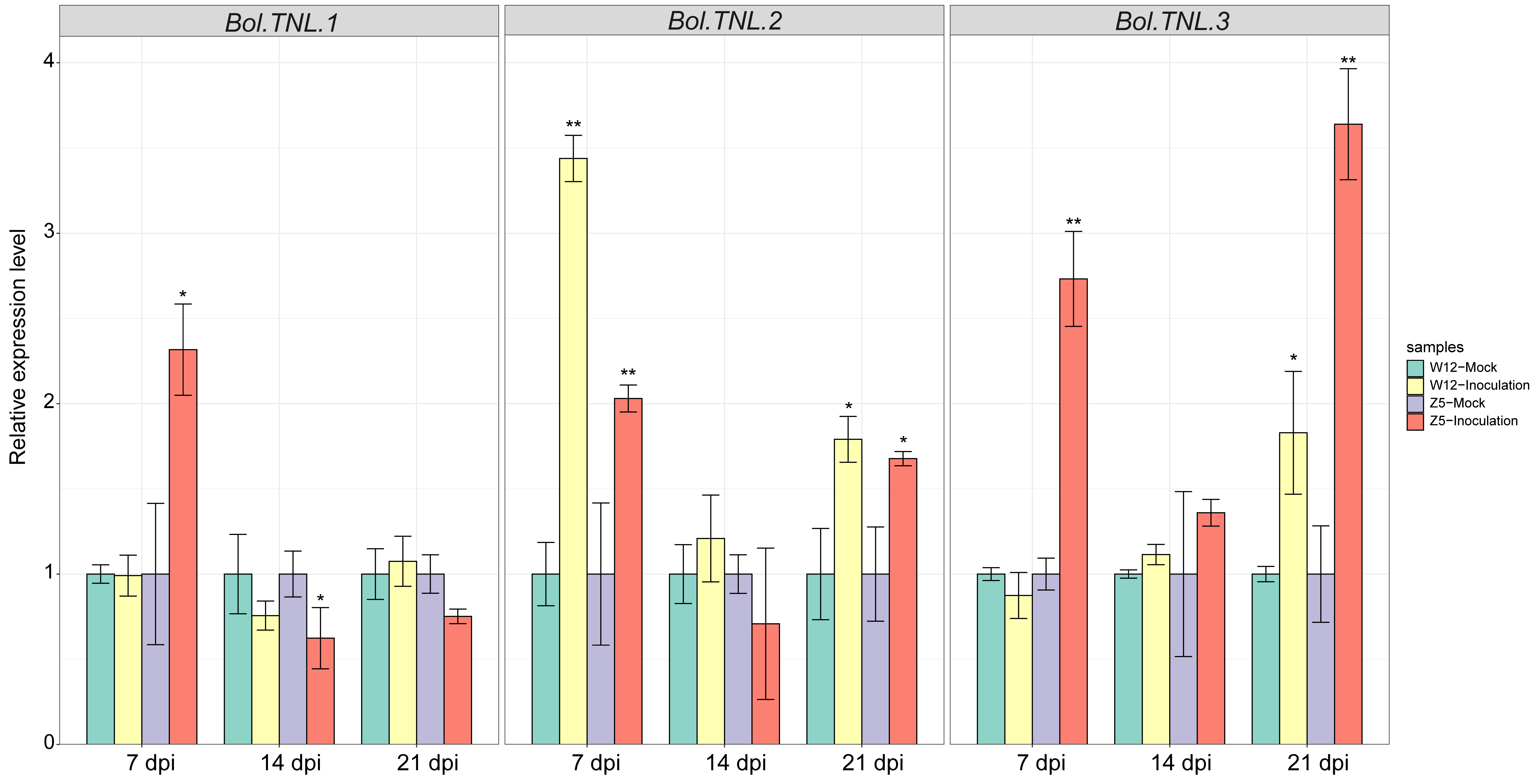

Supplement: Web_Material_uhaf208 [file web_material_uhaf208.zip › FigS4.tif]

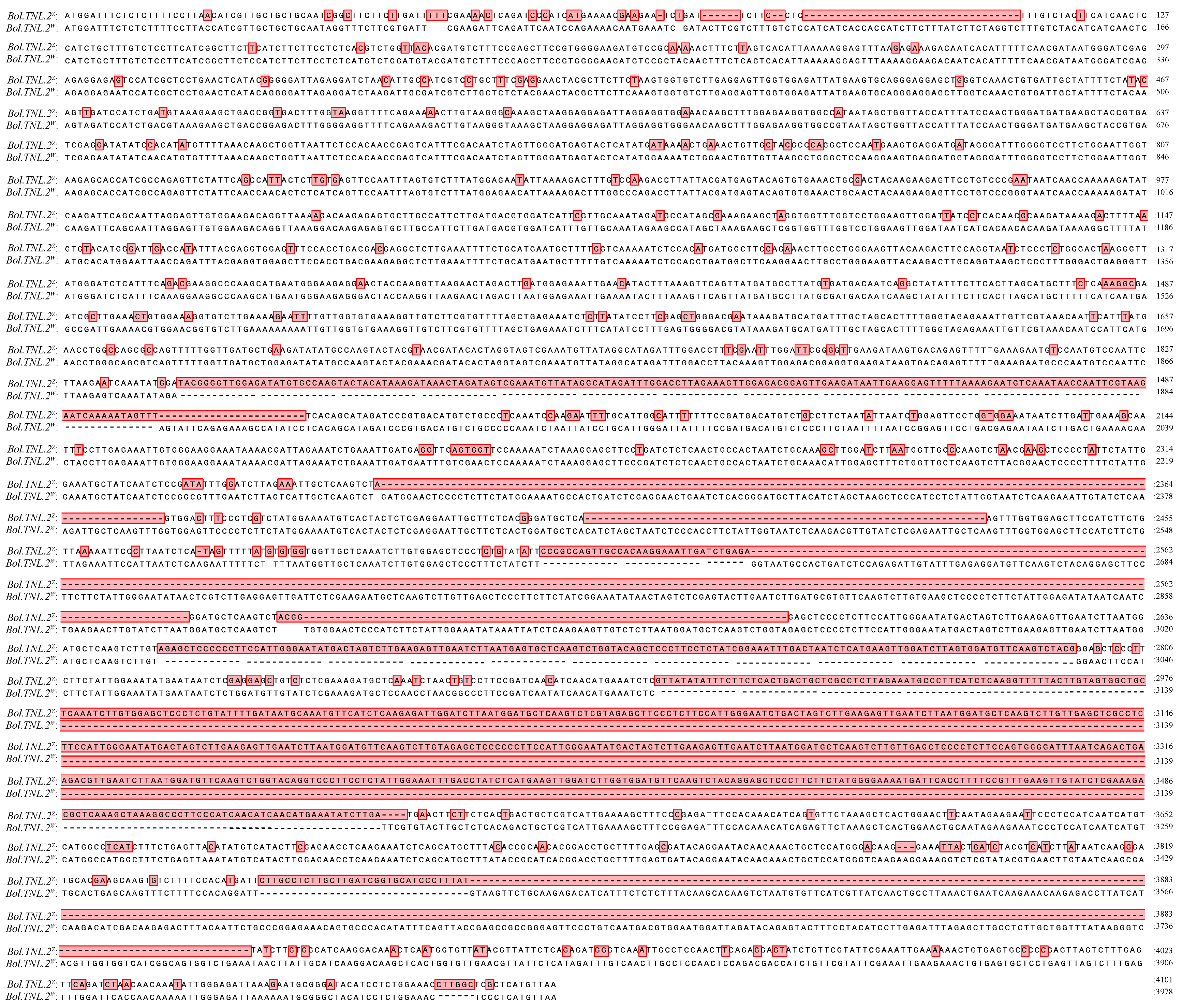

Supplement: Web_Material_uhaf208 [file web_material_uhaf208.zip › FigS5.tif]

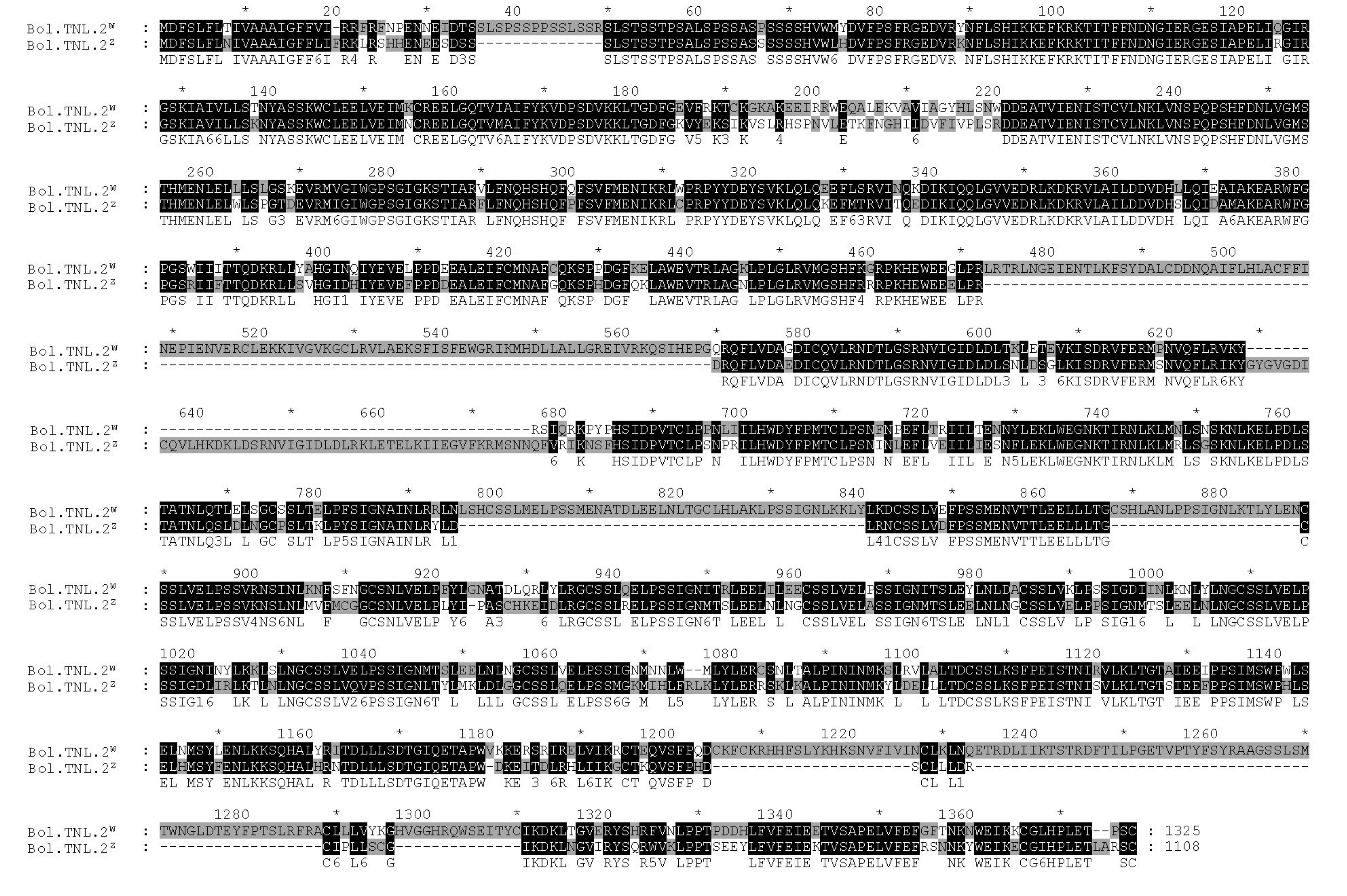

Supplement: Web_Material_uhaf208 [file web_material_uhaf208.zip › FigS6.tif]

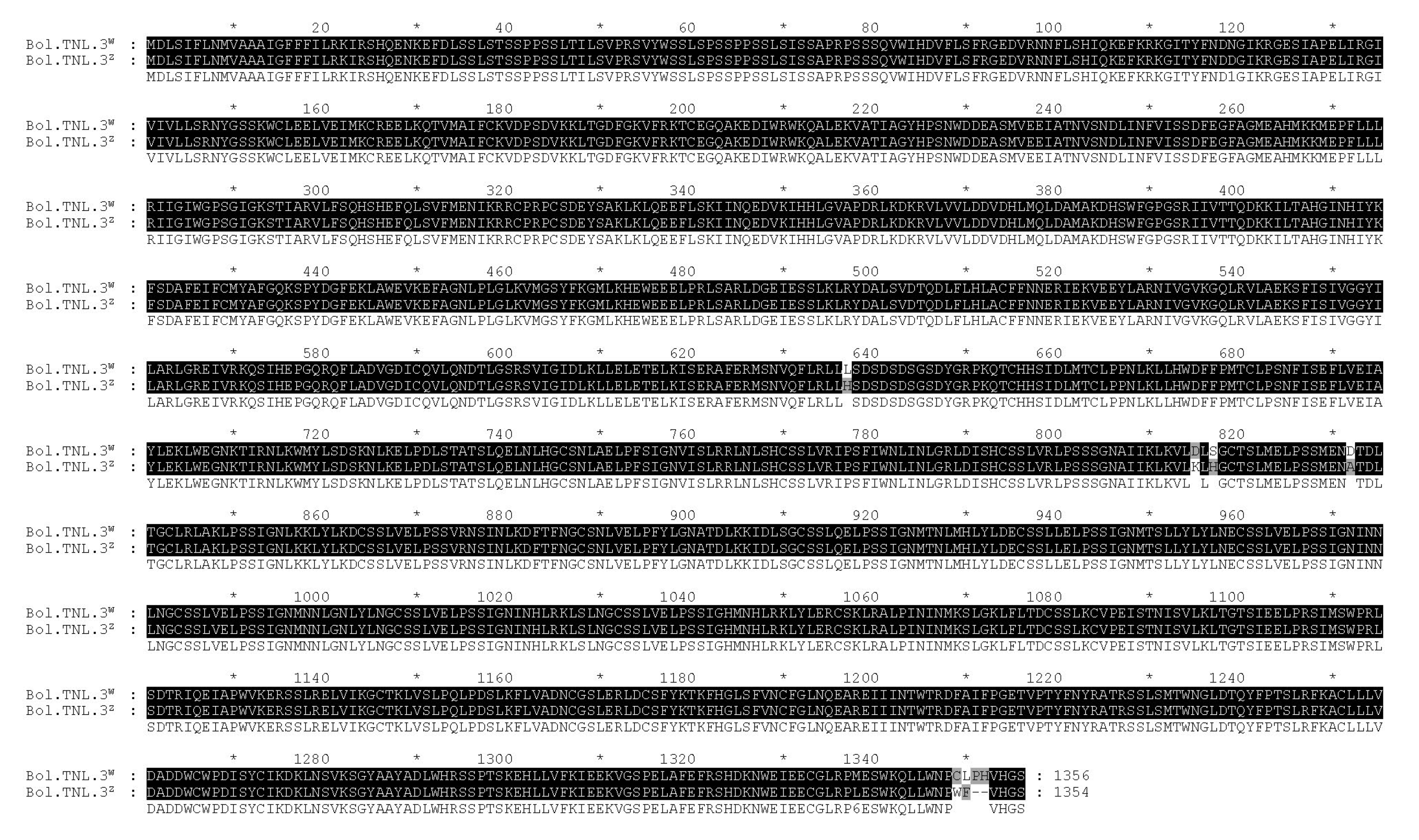

Supplement: Web_Material_uhaf208 [file web_material_uhaf208.zip › FigS7.tif]

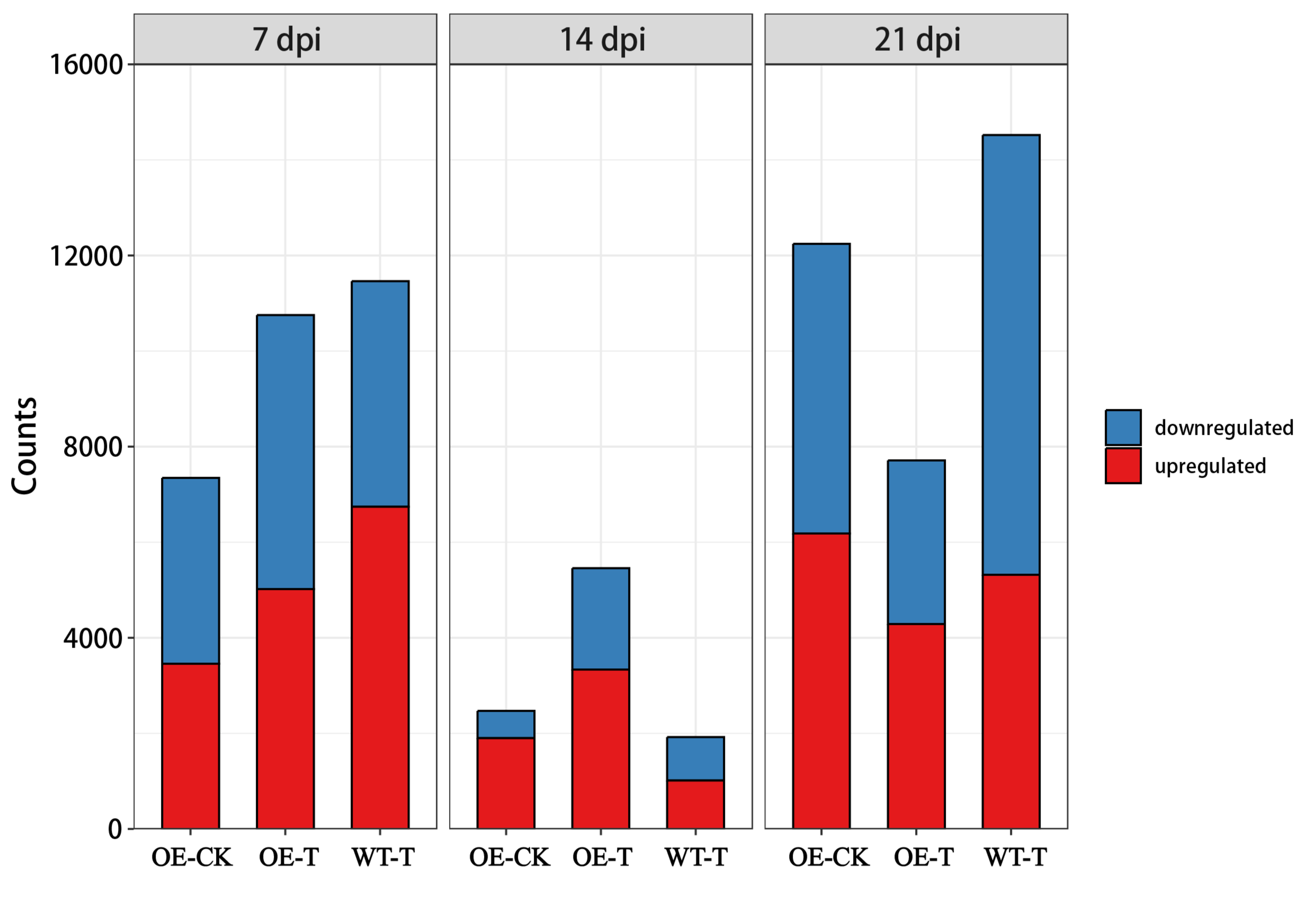

Supplement: Web_Material_uhaf208 [file web_material_uhaf208.zip › FigS8.tif]

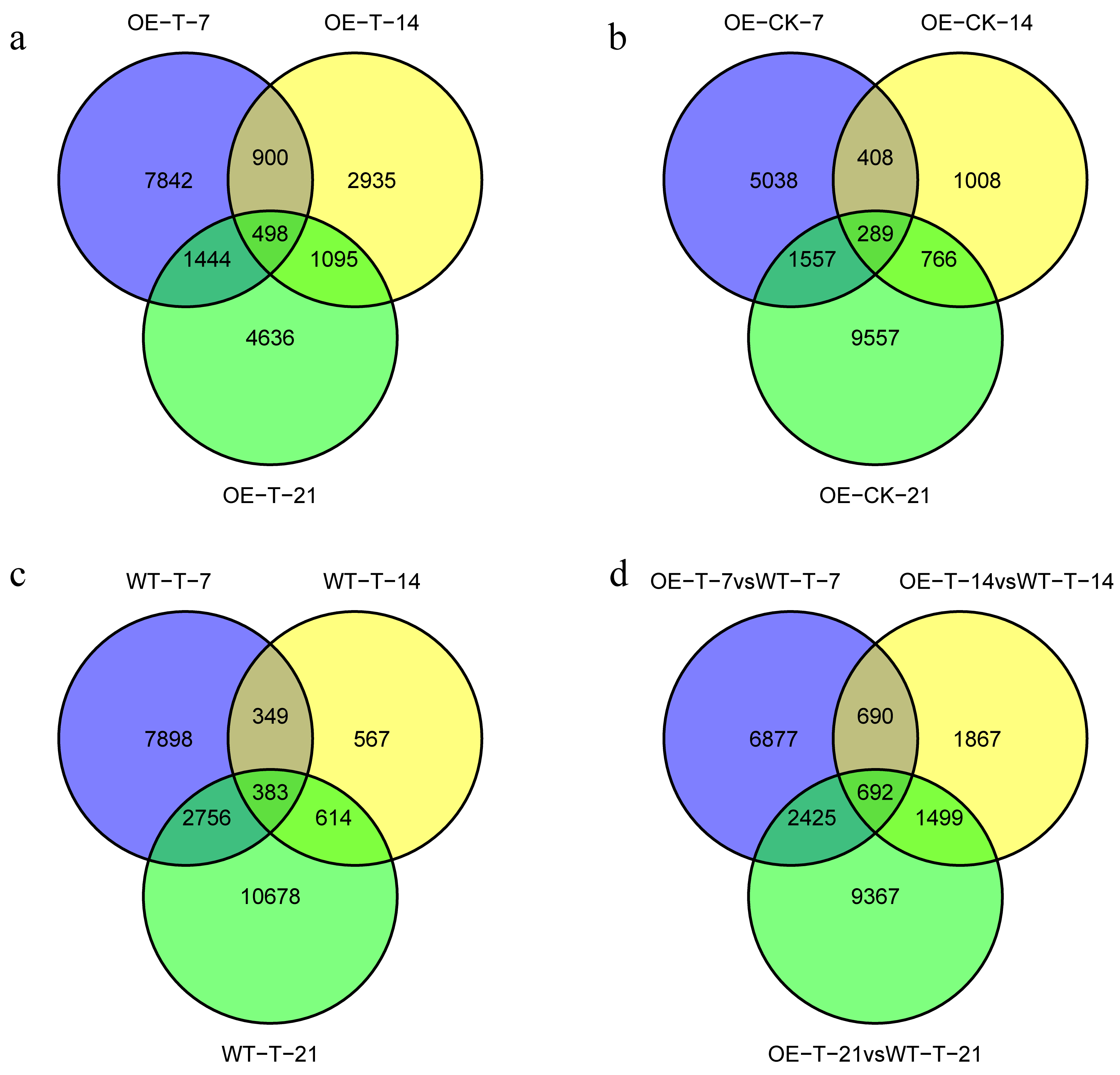

Supplement: Web_Material_uhaf208 [file web_material_uhaf208.zip › Figs9.tif]

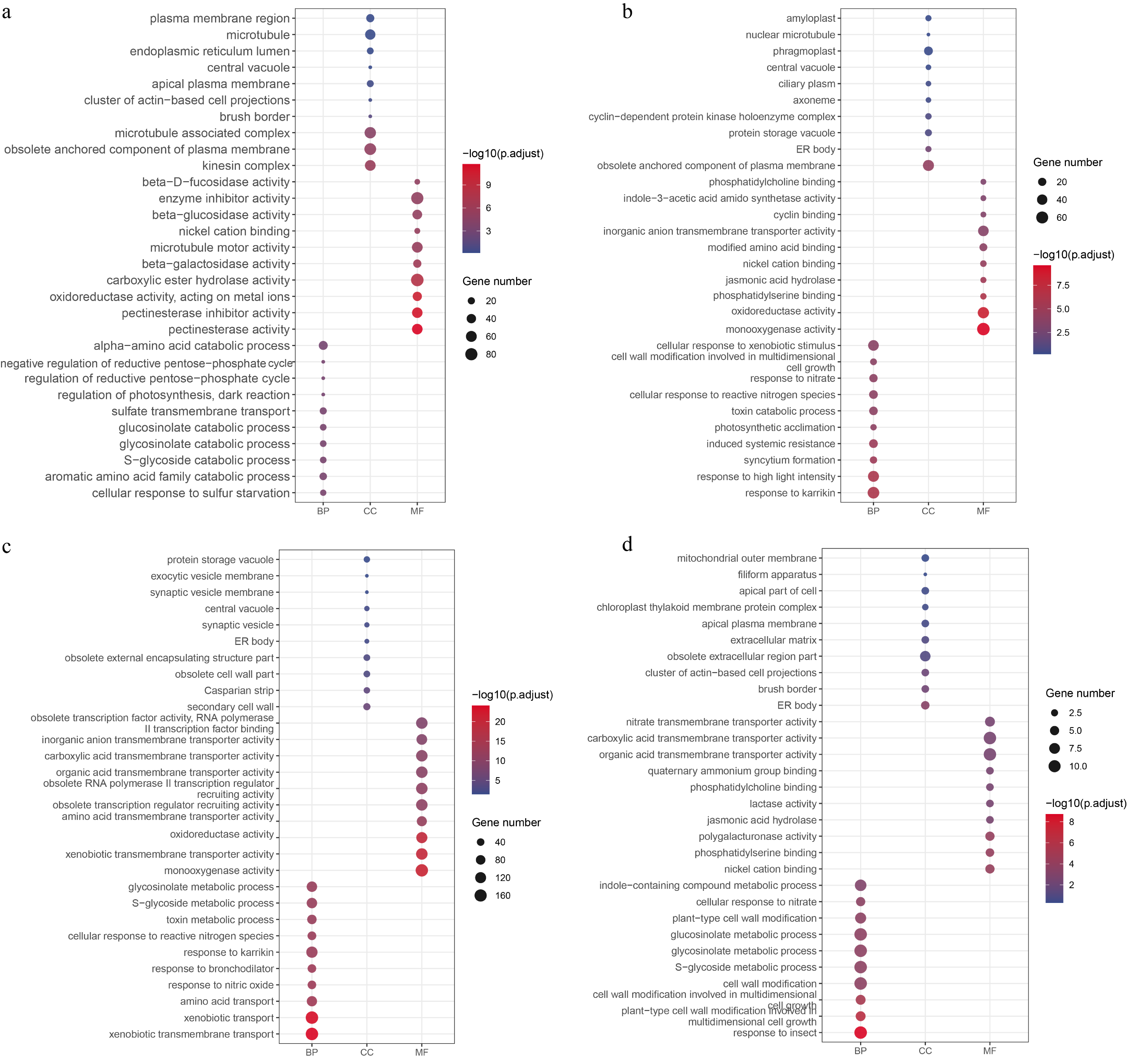

Supplement: Web_Material_uhaf208 [file web_material_uhaf208.zip › FigS10.tif]

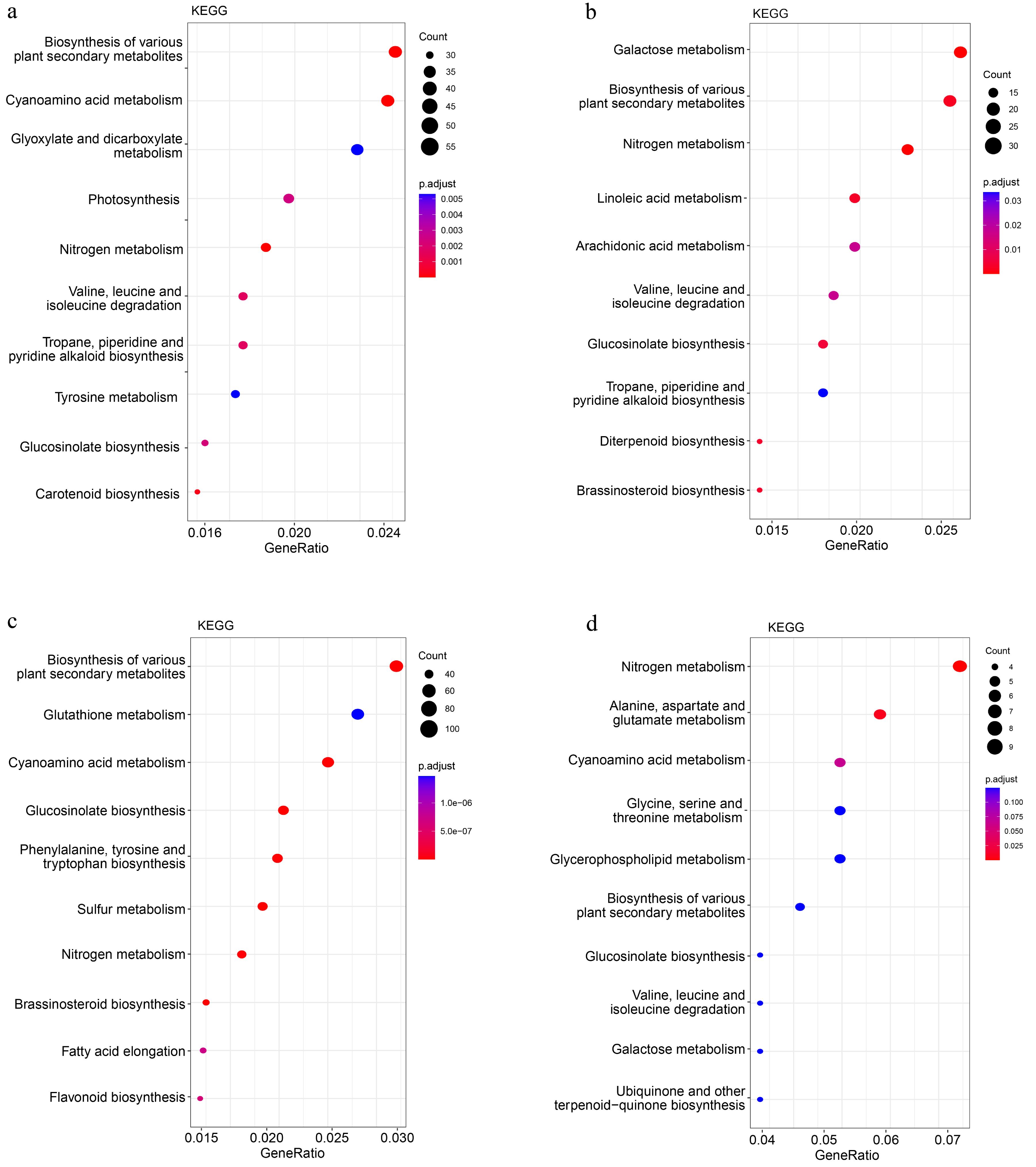

Supplement: Web_Material_uhaf208 [file web_material_uhaf208.zip › FigS11.tif]
